# Ectopic expression of *Jatropha curcas APETALA1* (*JcAP1*) caused early flowering in Arabidopsis, but not in Jatropha

**DOI:** 10.7717/peerj.1969

**Published:** 2016-04-25

**Authors:** Mingyong Tang, Yan-Bin Tao, Zeng-Fu Xu

**Affiliations:** 1 Key Laboratory of Tropical Plant Resources and Sustainable Use, Xishuangbanna Tropical Botanical Garden, Chinese Academy of Sciences, Menglun, Mengla, Yunnan, China; 2 College of Life Sciences, University of Chinese Academy of Sciences, Beijing, China

**Keywords:** Jatropha, Flower identity, Arabidopsis, *APETALA1*, Physic nut, Flowering

## Abstract

*Jatropha curcas* is a promising feedstock for biofuel production because Jatropha oil is highly suitable for the production of biodiesel and bio-jet fuels. However, Jatropha exhibits a low seed yield as a result of unreliable and poor flowering. *APETALA1* (*AP1*) is a floral meristem and organ identity gene in higher plants. The flower meristem identity genes of Jatropha have not yet been identified or characterized. To better understand the genetic control of flowering in Jatropha, an *AP1* homolog (*JcAP1*) was isolated from Jatropha. An amino acid sequence analysis of JcAP1 revealed a high similarity to the AP1 proteins of other perennial plants. *JcAP1* was expressed in inflorescence buds, flower buds, sepals and petals. The highest expression level was observed during the early developmental stage of the flower buds. The overexpression of *JcAP1* using the cauliflower mosaic virus (CaMV) 35S promoter resulted in extremely early flowering and abnormal flowers in transgenic Arabidopsis plants. Several flowering genes downstream of *AP1* were up-regulated in the *JcAP1*-overexpressing transgenic plant lines. Furthermore, *JcAP1* overexpression rescued the phenotype caused by the Arabidopsis AP1 loss-of-function mutant *ap1-11*. Therefore, *JcAP1* is an ortholog of *AtAP1,* which plays a similar role in the regulation of flowering in Arabidopsis. However, the overexpression of *JcAP1* in Jatropha using the same promoter resulted in little variation in the flowering time and floral organs, indicating that *JcAP1* may be insufficient to regulate flowering by itself in Jatropha. This study helps to elucidate the function of *JcAP1* and contributes to the understanding of the molecular mechanisms of flower development in Jatropha.

## Introduction

With the decreasing availability of fossil fuels and the deteriorating trend of environmental pollution, biodiesel has garnered significant attention as an alternative fuel (Mofijur et al., 2016). Physic nut (*Jatropha curcas* L.) is a perennial plant that belongs to the *Euphorbiaceae* family. Jatropha is monoecious, with male and female flowers borne on the same inflorescence (Divakara et al., 2010; Pandey et al., 2012; Wu et al., 2011). The potential benefit of growing Jatropha as a cash crop for biofuel in tropical and sub-tropical countries is now widely recognized (Akashi, 2012; Khalil et al., 2013; Pua et al., 2011). Jatropha has been propagated as a unique plant with biodiesel potential because of its multipurpose value, high oil content, high biomass productivity, adaptability to marginal land under a variety of agro-climatic conditions, and non-competitiveness with food production (Akashi, 2012; Khalil et al., 2013; Pandey et al., 2012; Pua et al., 2011). The oil content of Jatropha seeds and kernels ranges from 30 to 40% and 40 to 50% by weight, respectively (Pan & Xu, 2011; Sinha et al., 2015). Oil from Jatropha contains high levels of polyunsaturated fatty acids; therefore, Jatropha is suitable as a feedstock for the production of biodiesel and bio-jet fuel (Ong et al., 2011; Pramanik, 2003). The whole-genome sequence and genetic mapping of Jatropha have been reported (Hirakawa et al., 2012; Wu et al., 2015), and several genetic transformation methods have been established (Fu et al., 2015; Gu et al., 2015; Kajikawa et al., 2012; Kumar et al., 2010; Mao, Ye & Chua, 2013; Misra et al., 2012; Pan, Fu & Xu, 2010). Consequently, it is convenient to clone Jatropha genes and analyze their functions. However, the potential of Jatropha as a biofuel plant is limited by its low seed production (King et al., 2015). Despite the clear evidence of the abundant biomass generated by Jatropha, these data are not indicative of high seed productivity (Ghosh et al., 2007). Jatropha exhibits an overabundance of vegetative shoots and leaves in that could develop into reproductive shoots under suitable conditions. Thus, a reduction of undesired vegetative growth is imperative (Ghosh et al., 2010; Tjeuw, Slingerland & Giller, 2015). In addition, unreliable and poor flowering is an important factor that contributes to low seed productivity in Jatropha (Divakara et al., 2010). Therefore the elucidation of the genetic basis of flowering in Jatropha would be helpful for the molecular breeding of high-yielding Jatropha cultivars.

The *APETALA1* (*AP1*) was identified as a floral meristem identity gene to regulate flowering in many plant species. Mandel et al. (1992) reported that *AP1* encodes a putative transcription factor containing a MADS domain. This gene acts locally to specify the identity of the floral meristem and determine sepal and petal development. *AP1* and *LEAFY* (*LFY*) are pivotal in the switch to the reproductive phase. During floral initiation, a positive feedback loop between *AP1* and *LFY* is mediated by direct interactions (Kaufmann et al., 2010; Liljegren et al., 1999; William et al., 2004). After transition to flowering, the expression of *AP1* appears to be only indirectly affected by *LFY* (Wagner, Sablowski & Meyerowitz, 1999). The *AP1*, *FRUITFULL* (*FUL*) and *CAULIFLOWER* (*CAL*) genes act redundantly to control the flower meristem identity and inflorescence architecture by affecting *LFY* and *TFL1* expression levels (Ferrándiz et al., 2000). *AP1* and *CAL* are expressed in floral meristems and developing sepal and petal primordial cells (Blazquez et al., 2006; Mandel et al., 1992).

In Arabidopsis *ap1* mutants, the sepals are converted to bract-like structures, the petals are absent, the bract-like organs of the first whorl subtend secondary flowers in the second whorl, and tertiary flowers can also form (Bowman et al., 1993; Irish & Sussex, 1990; Mandel et al., 1992; Ng & Yanofsky, 2001). Recent studies have indicated that *AP1* can regulate cytokinin levels through the suppression of cytokinin biosynthesis and the activation of cytokinin degradation. These effects mediate the function of *AP1* in establishing determinate floral meristems in Arabidopsis (Han et al., 2014). In 35S:*AP1* Arabidopsis plants, extremely early flowering occurs after the production of five leaves, and the primary shoot meristem has been converted into a compound terminal flower. The secondary shoot meristems present in the axils of cauline leaves have been transformed into solitary flowers. In addition, 35S:*AP1* Arabidopsis can partially complement the later flowering phenotype of a *lfy* mutant (Liljegren et al., 1999). Constitutive expression of the Arabidopsis *AP1* gene in juvenile citrus seedlings resulted in transgenic citrus plants with fertile flowers and fruits after just one year of growth. The transgenic citrus exhibited an appreciably shortened juvenile phase (Peña et al., 2001). In Populus, the overexpression of the Populus ortholog of *APETALA1 (LAP1)* produced a novel function in photoperiodic regulation of seasonal growth, the *LAP1* overexpression resulted in severe attenuation of SD-mediated growth cessation in hybrid aspen (Azeez et al., 2014).

However, the function analysis of *AP1* gene in Jatropha has not been reported. Currently, only one flowering-related gene, Jatropha *FLOWERING LOCUS T* (*JcFT*), has been functionally analyzed in Jatropha (Li et al., 2014). Overexpression of *JcFT* can produce more seeds in a shorter time frame by shortening the flowering time in Jatropha, suggesting the possibility to increase seed yield by manipulating the flowering time (Chen et al., 2014). Therefore, in this study, we cloned and characterized a Jatropha *AP1* homolog, *JcAP1*, through genetic complementation of the Arabidopsis *AP1* loss-of-function mutant *ap1-11*. We analyzed the function of *JcAP1* in flowering induction and floral organ specification using transgenic Arabidopsis and Jatropha plants.

## Materials and Methods

### Plant materials and growth conditions

The roots, stems, mature leaves, inflorescence buds, flower buds, male flowers, female flowers and fruits of Jatropha were collected during the summer from Xishuangbanna, Yunnan Province, China. All of the tissues to be prepared for qRT-PCR were immediately frozen in liquid N_2_ and stored at −80°C until use. The wild-type (WT) *Arabidopsis thaliana* Columbia ecotype (Col-0) and the *ap1-11* mutant of the same ecotype were purchased from The Arabidopsis Information Resource (TAIR) website (http://www.arabidopsis.org/). The seeds of the Arabidopsis plants were germinated on 1/2 MS medium for one-week. Then, the seedlings were transferred to peat soil in plant growth chambers maintained at 22 ± 2 °C under long-day (16 light/8 h dark) or short-day (8 light/16 h dark) conditions. Phenotype analysis was performed on homozygous (T2) Arabidopsis plants and heterozygous (T0) Jatropha plants. More than 20 plants were used for the characterization of each Arabidopsis genotype. The number of rosette leaves and the number of days between transplantation to soil and appearance of the first visible flower bud were recorded. The aboveground tissues of 15-day-old Arabidopsis seedlings were harvested to analyze mRNA transcription levels.

### Cloning of JcAP1 cDNA

Total RNA was extracted from the Jatropha flowers using the protocol described by Ding et al. (2008). First-strand cDNA was synthesized using M-MLV-reverse transcriptase according to the manufacturer’s instructions (TAKARA, Dalian, China). The full-length *JcAP1* genomic DNA sequence (Sato et al., 2011) (http://www.kazusa.or.jp/jatropha/) was amplified via PCR using the primers XK928 and XK929 (Table S1), which introduced *Kpn*I and *Sal*I recognition sites, respectively. The *JcAP1* cDNA containing full-length coding sequence was amplified from flower cDNA with the same primers. The PCR products were subsequently cloned into the pGEM-T vector (Promega Corporation, Madison, Wisconsin, USA) and sequenced. All primers used in this research were listed in Table S1.

### Sequence and phylogenetic analyses

JcAP1 amino acid sequence was deduced according to the coding sequence. Related sequences were identified through a BLAST search (http://www.ncbi.nlm.nih.gov/BLAST/). To determine the amino acid identities, the alignment results were subjected to pairwise comparisons using DNAMAN 6.0. A phylogenetic tree based on the protein sequences was constructed with MEGA 5.0 (http://www.megasoftware.net). The amino acid sequences of AP1s, FULs, and CALs were assembled using ClustalX. A neighbor-joining phylogenetic tree was generated with MEGA 5.0 using the Poisson model, with gamma-distributed rates and 1,000 bootstrap replicates.

### Plant expression vector construction and Arabidopsis and Jatropha transformation

To construct the 35S:*JcAP1* plant overexpression vector, the *JcAP1* sequence was excised from the pGEM-T vector (Promega, Corporation, Madison, Wisconsin, USA) using the restriction enzymes *Kpn*I and *Sal*I. Next, *JcAP1* was cloned into the pOCA30 vector containing the CaMV 35S promoter. Transformation of Arabidopsis WT and *ap1-11* mutant plants with the *Agrobacterium* strain EHA105 carrying the 35S:*JcAP1* construct was performed using the floral dip method (Clough & Bent, 1998). Transformation of Jatropha with the *Agrobacterium* strain EHA105 carrying the same construct was performed according to the protocol described by Pan, Fu & Xu (2010) and Fu et al. (2015). All of the transgenic plants were confirmed using genomic PCR and RT-PCR.

### Expression analysis via quantitative RT-PCR (qRT-PCR)

The roots, stems, mature leaves, inflorescence buds, flower buds, male flowers, female flowers and fruits of mature Jatropha plants and the aboveground tissues of 15 days Arabidopsis seedlings were collected for qRT-RCR detection. Total RNA was extracted from frozen Jatropha tissues as described by Ding et al. (2008). Total RNA was extracted from frozen Arabidopsis tissues using TRIzol reagent (Transgene, China). First-strand cDNA was synthesized with the PrimeScript^®^ RT Reagent Kit with gDNA Eraser (TAKARA, Dalian, China). The cDNA templates of first-strand cDNA were diluted 5-fold with sterilized double-distilled water. qRT-PCR was performed using SYBR^®^ Premix Ex Taq™ II (TAKARA, Dalian, China) on a Roche 480 Real-Time PCR Detection System (Roche, Mannheim, Germany). The primers employed for qRT-PCR are listed in Table S1. qRT-PCR was conducted with three independent biological replicates and three technical replicates for each sample. The data were analyzed using the 2${}^{-\Delta \Delta \mathrm{CT}}$ method described by Livak & Schmittgen (2001). The transcript levels of specific genes were normalized using Jatropha *ACTIN1* or Arabidopsis *ACTIN2*.

## Results

### Cloning and sequence analysis of *JcAP1*

A combined reverse transcriptase-polymerase chain reaction (RT-PCR) strategy was used to isolate *AP1*-like cDNA (*JcAP1*) from Jatropha. The *JcAP1* coding sequence (CDS) (GenBank accession no. KR013222) is comprised of 732 bp and encodes a 243-amino acid protein showing 81%, 79%, 75%, and 71% sequence identity to *Vitis vinifera* VvAP1 (Calonje et al., 2004), *Populus trichocarpa* PtAP1 (Tuskan et al., 2006), *Coffea arabica* CaAP1 (De Oliveira et al., 2014), and AtAP1 (Mandel et al., 1992), respectively.

**Figure 1:**
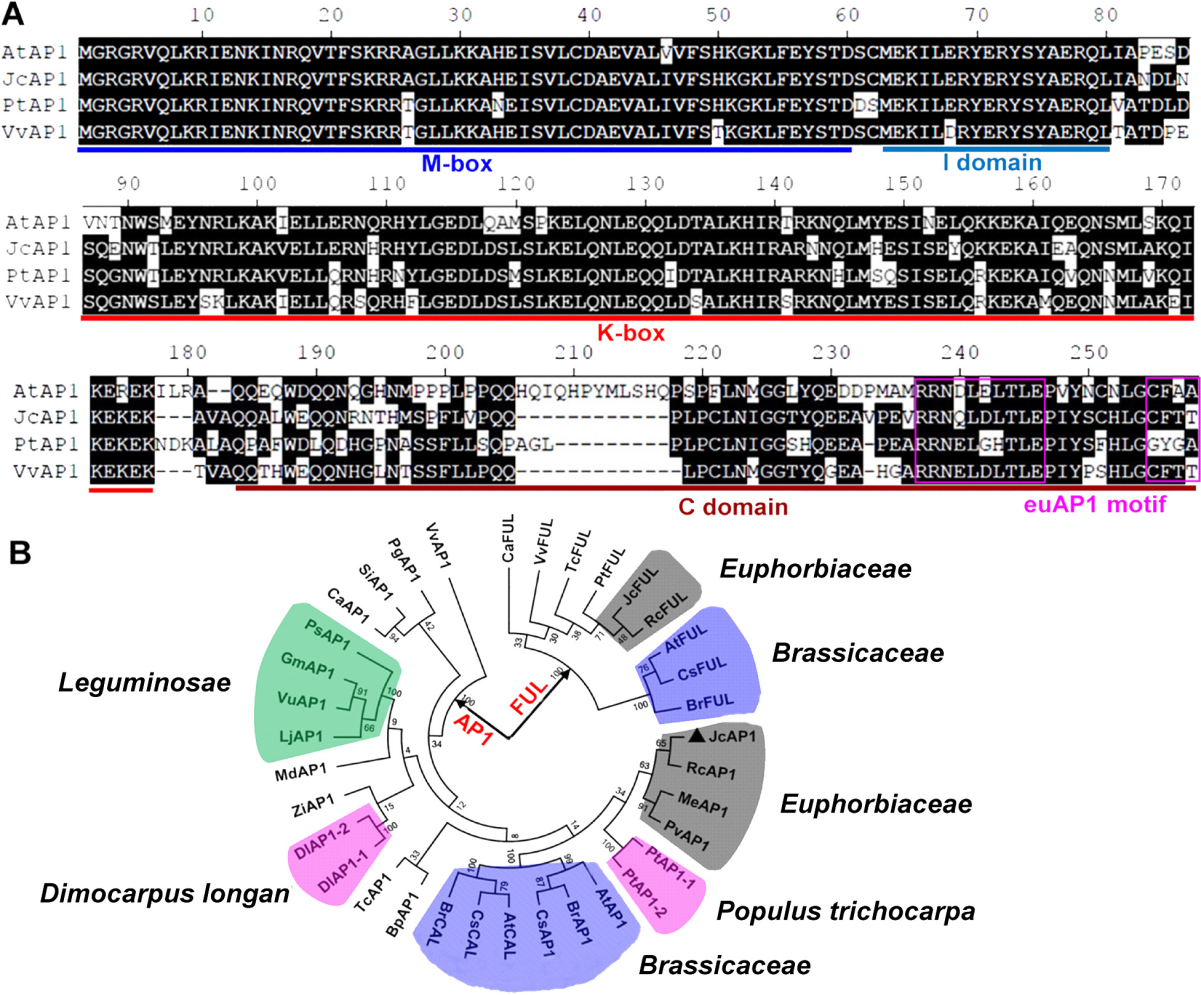
Comparison and phylogenetic analysis of *JcAP1* and other *AP1* genes. (A) Sequence alignment of the JcAP1, AtAP1, PtAP1, and VvAP1 amino acid sequences. Similar amino acid residues are shaded in black. Dots denote gaps. Each colored line under the alignment indicates a different domain of an AP1 homolog. “M-box” indicates the highly conserved MADS-box domain; “I domain” indicates the intervening domain; “K-box” indicates the conserved keratin-like domain; “C domain” indicates the C-terminal domain; and “euAP1 motif” indicates the euAP1 motif. (B) Phylogenetic analysis of AP1 homologs from different plant species: AtAP1, *Arabidopsis thaliana AP1 ( NP_177074); BpAP1, Betula platyphylla* AP1 ( AFV92462); BrAP1, *Brassica rapa AP1 ( XP_009105460.1); DlAP1-1, Dimocarpus longan* AP1-1 ( AEZ63951); DlAP1-2, *Dimocarpus longan AP1-2 ( AGC13077); JcAP1, Jatropha curcas AP1 ( KR013222); PtAP1-1, Populus trichocarpa* AP1-1 ( XP_002311353); PtAP1-2, *Populus trichocarpa AP1-2 ( XP_002316076); RcAP1, Ricinus communis AP1 ( XP_002514623); CaAP1, Coffea arabica* AP1 ( AHW58038); MdAP1, *Malus domestica AP1 ( ACD69426)*; SiAP1, *Sesamum indicum AP1 ( AIS82596); VvAP1, Vitis vinifera* AP1 ( NP_001268210); PgAP1, *Panax ginseng AP1 ( BAK20019); LjAP1, Lotus japonicus* AP1 ( AAX13296); VuAP1, *Vigna unguiculata AP1 ( BAJ22385); CsAP1, Camelina sativa* AP1 ( XP_010415539); TcAP1, *Theobroma cacao AP1* ( XP_007045796); PvAP1, *Plukenetia volubilis AP1*( KU942379); MeAP1, *Manihot esculenta AP1(_029935m http://treetfdb.bmep.riken.jp); PsAP1, Pisum sativum* AP1 ( AAL66379); GmAP1, *Glycine max AP1 ( XP_003531957); ZjAP1, Ziziphus jujube* AP1 ( ACG70964); AtCAL, Arabidopsis thaliana *CAL ( NP_564243); BrCAL, Brassica rapa* CAL ( XP_009109914); CsCAL, *Camelina sativa CAL ( XP_010477869); AtFUL, Arabidopsis thaliana* FUL ( NP_568929); CaFUL, *Coffea arabica FUL ( AHW58040); JcFUL, Jatropha* curcas *FUL ( KDP31379); PtFUL, Populus trichocarpa* FUL ( ABK92820); VvFUL, *Vitis vinifera ( XP_002263017); RcFUL, Ricinus communis* FUL ( KDP31379); BrFUL, Brassica rapa *FUL ( XP_009130138); CsFUL, Camelina sativa* FUL ( XP_010443902); TcFUL, *Theobroma cacao* FUL ( XP_007037634); The phylogeny of these AP1 homologs was determined based on their amino acid sequences using MEGA5 and the neighbor-joining method. Bootstrap values were obtained using 1,000 bootstrap replicates.

The genomic sequence of *JcAP1* was 4,928 bp and consisted of eight exons and seven introns, which resembles the genomic structure of the Arabidopsis *AP1* gene (Mandel et al., 1992). Multiple alignments were performed using the JcAP1 sequence and the sequences of AP1 homologs from other species. The MADS-box domain, K-Box domain, and euAP1 motif were determined (Fig. 1A). Since AP1 is closely related to CAL and FUL, and three genes exhibit high similarity and share redundant functions for floral meristem specification (Bowman et al., 1993), we undertook a phylogenetic analysis of the AP1/FUL MADS-box gene lineage (Fig. 1B), which is also called the *SQUA* lineage (Krogan & Ashton, 2000). The phylogenetic tree is divided into two clades, AP1 clade and FUL clade. CAL, which appears only in *Brassicaceae*, is clustered in the AP1 clade (Litt & Irish, 2003). Because CAL and AP1 originated from a recent duplication event <60 million years ago (Alvarez-Buylla, Garcia-Ponce & Garay-Arroyo, 2006). JcAP1 isolated in this study is clustered in the AP1 clade, while the JcFUL is clustered in the FUL clade. Moreover, JcAP1 is more closely related to AP1s of the *Euphorbiaceae* plants, such as *Ricinus communis* RcAP1, *Manihot esculenta* MeAP1 and *Plukenetia volubilis* PvAP1. In addition, the JcFUL is also closely related to *Ricinus communis* RcFUL (Fig. 1B). The results indicated that the AP1/FUL MADS-box gene phylogeny follows species phylogeny to interact with other MADS box proteins to confer sepal and petal identity (Mandel et al., 1992).

### Expression pattern of *JcAP1* in Jatropha

**Figure 2:**
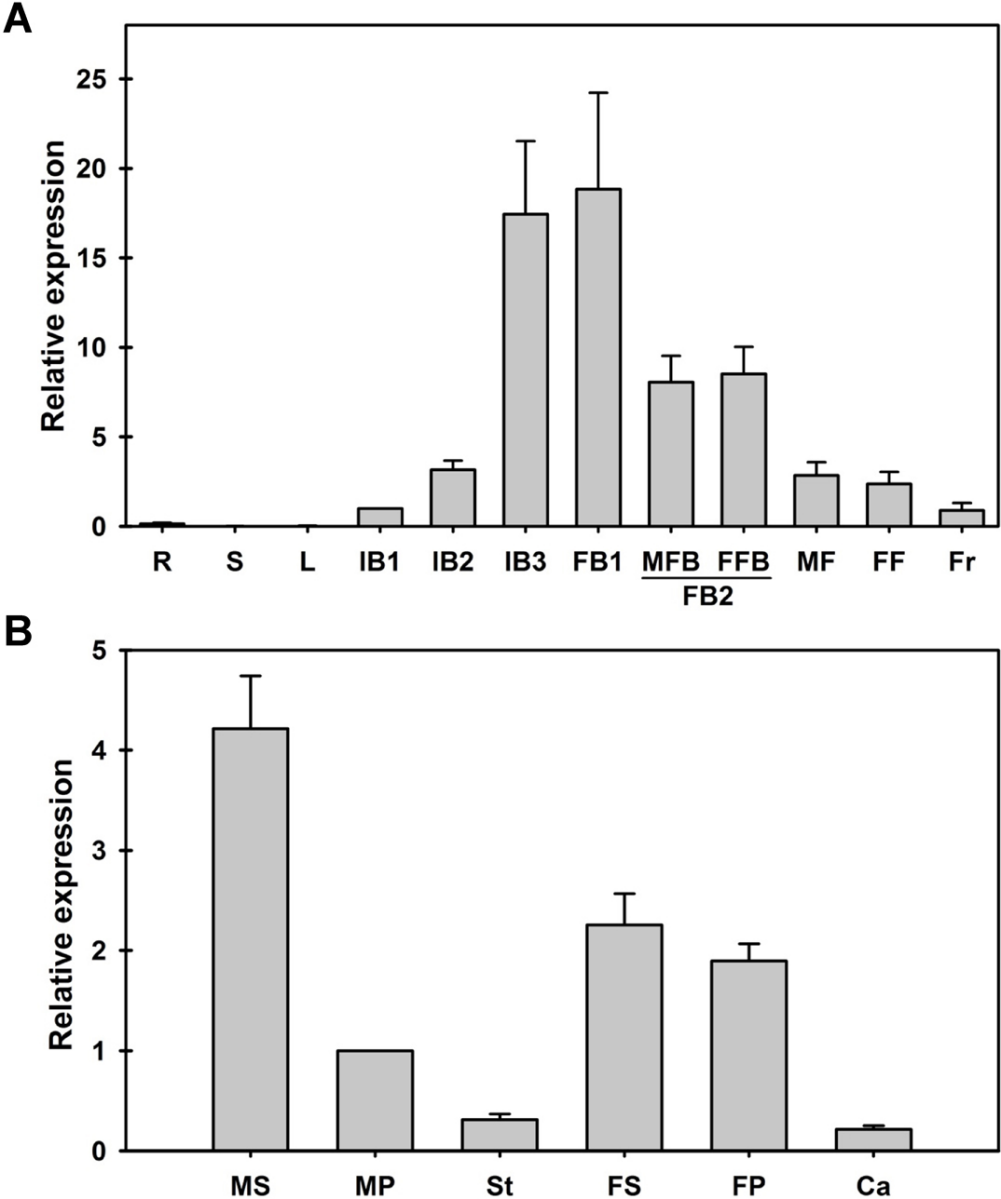
Expression of *JcAP1* in various adult Jatropha organs. (A) The expression level of *JcAP1* in the roots, stems, leaves, inflorescence buds, flower buds, flowers and fruits. (B) The expression level of *JcAP1* in different whorls of male and female flowers. The qRT-PCR results were obtained from two independent biological replicates and three technical replicates for each sample. The error bars represent the standard deviation. R, roots; S, stems; L, mature leaves; IB1, inflorescence bud stage 1 (0–5 days, inflorescence buds are visible); IB2, inflorescence bud stage 2 (1 week after IB1); IB3, inflorescence bud stage 3 (1 week after IB2); FB1, flower bud stage 1 (1 week after IB3); FB2, flower bud stage 2 (1 week after FB1, the male flower bud (MFB) and female flower bud (FFB) are identifiable); MF, male flower (1 week after MFB); FF, female flower (1 week after FFB). Fruits (Fr) were harvested 15 days after fertilization. Male sepals (MS), male petals (MP), stamens (St), female sepals (FS), female petals (FP), and carpels (Ca) were harvested 1 or 2 days before the male and female flowers bloomed. The levels of the detected amplicons were normalized using the amplified product of *JcACTIN1*. The mRNA levels in the IB1 and male petal tissues were used as standards, with a set value of 1.

To assess the expression pattern of *JcAP1* in Jatropha, we performed a qRT-PCR analysis using RNA extracted from the roots, stems, mature leaves, inflorescence buds, flower buds, male flowers, female flowers and fruits. The morphologies of different developmental stages of flower were shown in Fig. S3. *JcAP1* was expressed in the inflorescence buds, flower buds, flowers, and fruits but showed very low expression in the roots, shoots and leaves. The expression profiles revealed that *JcAP1* was highly expressed during the later stages of inflorescence buds (IB3) and early stages of flower buds (FB1). During the development of inflorescences, the expression levels of *JcAP1* increased, whereas *JcAP1* expression decreased during the development of flower organs. In the reproductive organs, the fruits showed the lowest expression level of *JcAP1* (Fig. 2A). *JcAP1* was expressed in all floral organs, particularly in the sepals and petals (Fig. 2B).

### Constitutive overexpression of *JcAP1* in Arabidopsis induces early flowering and abnormal flowers

**Figure 3:**
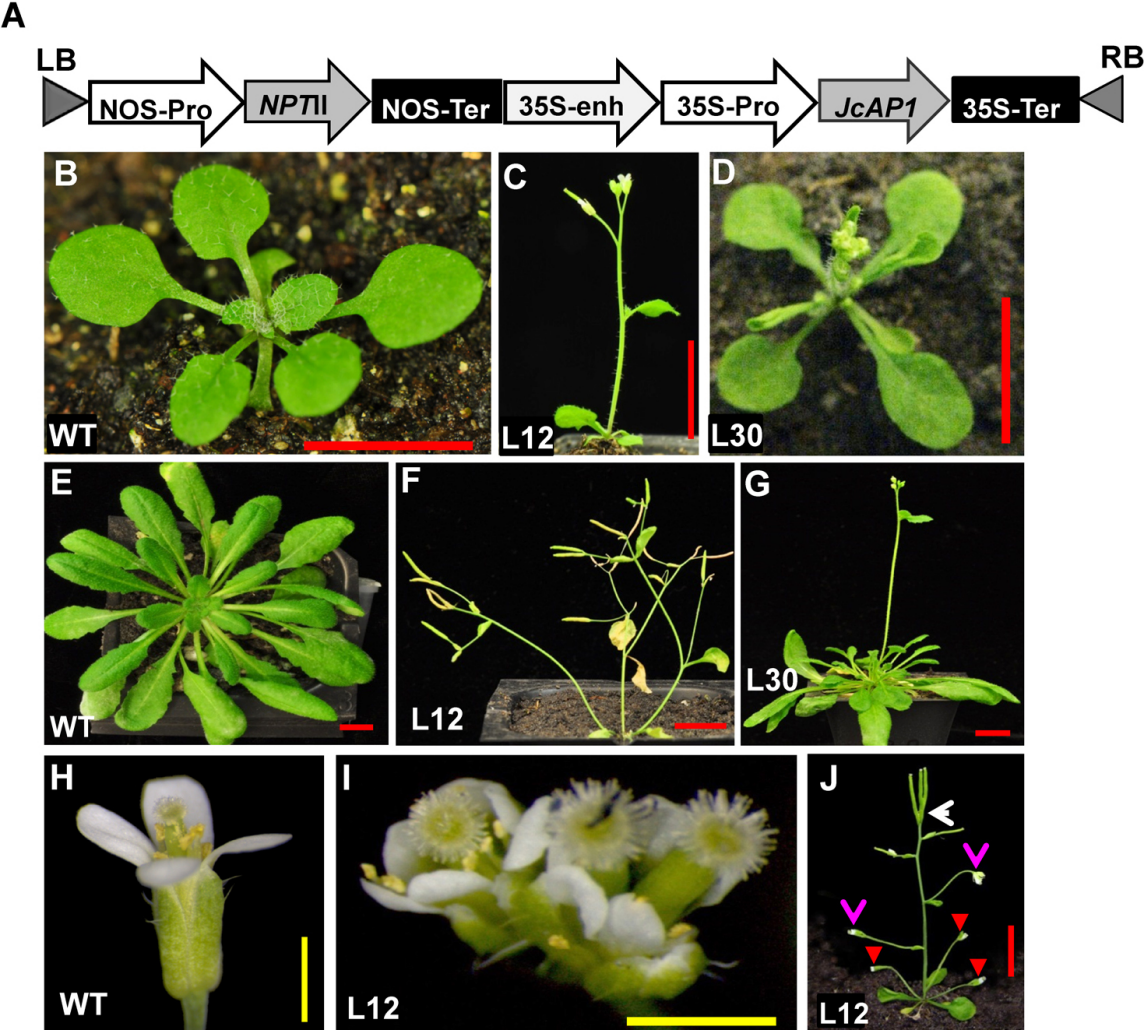
Ectopic expression of *JcAP1* results in early flowering and abnormal flowers in transgenic Arabidopsis. (A) Schematic diagram of the T-DNA region of the binary plasmid used in this study. NOS-Pro, nopaline synthase promoter; 35S-Pro, CaMV 35S promoter; 35S-Enh, CaMV 35S enhancer; NOS-Ter, nopaline synthase terminator; CaMV 35S-Ter, 35S terminator; RB, right border of the T-DNA region; and LB, left border of the T-DNA region. (B–D) 15-day-old seedlings grew under LD conditions. (E–G) 60-day-old seedlings grew under SD conditions. (H) A normal flower of a wild-type plant. (I) Abnormal flowers of 35S:*JcAP1* transgenic plants from L12. (J) A solitary flower appeared at the rosette and cauline leaf axils in the transgenic plants. Red arrows indicate the solitary rosette flowers; pink arrows indicate that primary shoots immediately terminated with the formation of a solitary flower; the white arrow indicates fruit formation of the abnormal terminal flowers. (B, E, and H) WT; (C, F, I, and J) transgenic L12; (D and G) transgenic Arabidopsis L30. Red bars = 1 cm, yellow bars = 1 mm.

To determine whether *JcAP1* is involved in the regulation of flowering time, *JcAP1* cDNA driven by the CaMV 35S promoter (Fig. 3A) was transformed into WT Arabidopsis. WT plants under the same growth conditions were used as a control. Transgenic plants were confirmed via qRT-PCR analysis of *JcAP1* expression using the aboveground tissues of 15-day-old Arabidopsis seedlings. Thirty-four independent T0 transgenic lines were generated with the 35S:*JcAP1* construct. Transgenic plants showed high *JcAP1* expression level (Fig. S1A). In the majority of the transgenic lines, bolting occurred notably earlier than in WT plants under both long-day (LD) and short-day (SD) conditions.

We examined the phenotypes of two independent homozygous transgenic lines (L12 and L30) in the T2 generation. Arabidopsis plants ectopically expressing *JcAP1* bolted 6–10 days earlier and produced 4–6 fewer rosette leaves than WT plants under LD conditions (Figs. 3B–3D and Table 1). Under SD conditions, Arabidopsis plants ectopically expressing *JcAP1* flowered approximately 1–2 months earlier than WT (Figs. 3E–3G and Table 2). Therefore, the overexpression of *JcAP1* in Arabidopsis significantly reduced the vegetative growth time.

**Table 1:** Overexpression of *JcAP1* promotes flowering in Arabidopsis under LD conditions.

Lines	*N*	Rosette leaves	Flower bud formation time(day)
WT	22	10.14 ± 0.89	20.18 ± 0.73
Line 12	20	3.95 ± 0.51^**^	10.60 ± 1.23^**^
Line 30	20	5.40 ± 0.99^**^	14.25 ± 1.37^**^

**Notes.**

WT plants and two independent *JcAP1*-overexpressing lines (L12 and L30) grown under LD conditions (16 h light/8 h dark) were subjected to the analysis of rosette leaves and flowering times. *N* = plant number. The rosette leaves and flowering times are presented as the mean ± standard deviation.

** Significantly different from the control at the 1% level.

**Table 2:** Overexpression of *JcAP1* promotes flowering in Arabidopsis under SD conditions.

Lines	*N*	Rosette leaves	Flower bud formation time(day)
WT	22	60.41 ± 3.95	104.00 ± 5.83
Line 12	25	14.68 ± 1.44^**^	33.72 ± 3.06^**^
Line 30	27	31.63 ± 2.50^**^	58.33 ± 3.95^**^

**Notes.**

WT plants and two independent *JcAP1*-overexpressing lines (L12 and L30) grown under SD growing conditions (8 h light/16 h dark) were subjected to the analysis of rosette leaves and flowering times. *N* = plant number. The rosette leaves and flowering times are presented as the mean ± standard deviation.

** Significantly different from the control at the 1% level.

In contrast to WT plants, the primary shoots of the transgenic plants were converted into compound terminal flowers consisting of two or three pistils surrounded by an abnormal number of sepals, petals, and stamens (Fig. 3I). Furthermore, the secondary shoots produced in cauline and rosette leaf axils were converted into solitary flowers. In extreme transgenic plants, all branches and inflorescences were replaced by solitary flowers (Fig. 3J).

Further analysis indicated that the promotion of flowering and abnormal terminal flowers in 35S:*JcAP1* transgenic Arabidopsis was correlated with a significant up-regulation of the floral meristem identity genes *AtLFY*, *AtFUL, AtAP1* and *AtCAL* and the floral organ identity genes *AtAGAMOUS* (*AtAG*), *AtAP3* and *AtSEPs* (*AtSEP1, AtSEP2, AtSEP3*) (Fig. S1). The expression levels of these genes were highest in transgenic plants L12, whereas the *AtTFL1* expression level was slightly down-regulated (Fig. S1). Thus, L12 also showed the most obvious changes in phenotype of extremely early flowering and solitary and terminal flowers (Fig. 3). Thus, the phenotypes of the early-flowering and abnormal terminal flowers produced due to the ectopic expression of *JcAP1* in transgenic Arabidopsis were similar to those resulting from *AtAP1* overexpression (Mandel & Yanofsky, 1995).

### Constitutive overexpression of *JcAP1* in *ap1-11* mutant Arabidopsis induces early flowering and partially complements the phenotype

To further determine whether *JcAP1* can function similarly to *AtAP1*, the 35S:*JcAP1* construct (Fig. 3A) was transformed into Arabidopsis *ap1-11* mutant plants. Eight independent T0 transgenic lines were generated and confirmed through qRT-PCR analysis of *JcAP1* expression using RNA from aboveground tissues of 15-day-old Arabidopsis seedlings. WT and *ap1-11* mutants under the same growth conditions were used as controls. Most of the transgenic lines bolted earlier than the WT and *ap1-11* mutant plants under inductive LD conditions. The *ap1* mutants didn’t exhibit significantly later flowering than WT (Table 3), which is because three homologous genes *AP1*, *CAL*, and *FUL* play redundant roles in control of flowering time. Each single mutant of *ap1, cal* or *ful* exhibited only slightly late flowering, whereas the triple mutant exhibited significantly late flowering (Ferrándiz et al., 2000).

**Table 3:** Overexpression of *JcAP1* in *ap1-11* Arabidopsis plants promotes flowering time under LD conditions.

Lines	*N*	Rosette leaves	Flower bud formation time(day)
WT	25	10.54 ± 0.92	20.18 ± 0.73
*ap1-11*	20	11.02 ± 0.87	20.80 ± 1.23
Line C2	15	8.40 ± 0.99^**^	14.05 ± 1.45^**^
Line C5	15	3.95 ± 0.51^**^	9.80 ± 1.18^**^

**Notes.**

WT plants, the *ap1-11* mutant, and two independent *JcAP1*-overexpressing lines (C2 and C5) grown under LD growing conditions (16 h light/8 h dark) were subjected to the analysis of rosette leaves and flowering times. *N* = plant number. The rosette leaves and flowering times are presented as the mean ± standard deviation.

** Significantly different from the control at the 1% level.

**Figure 4:**
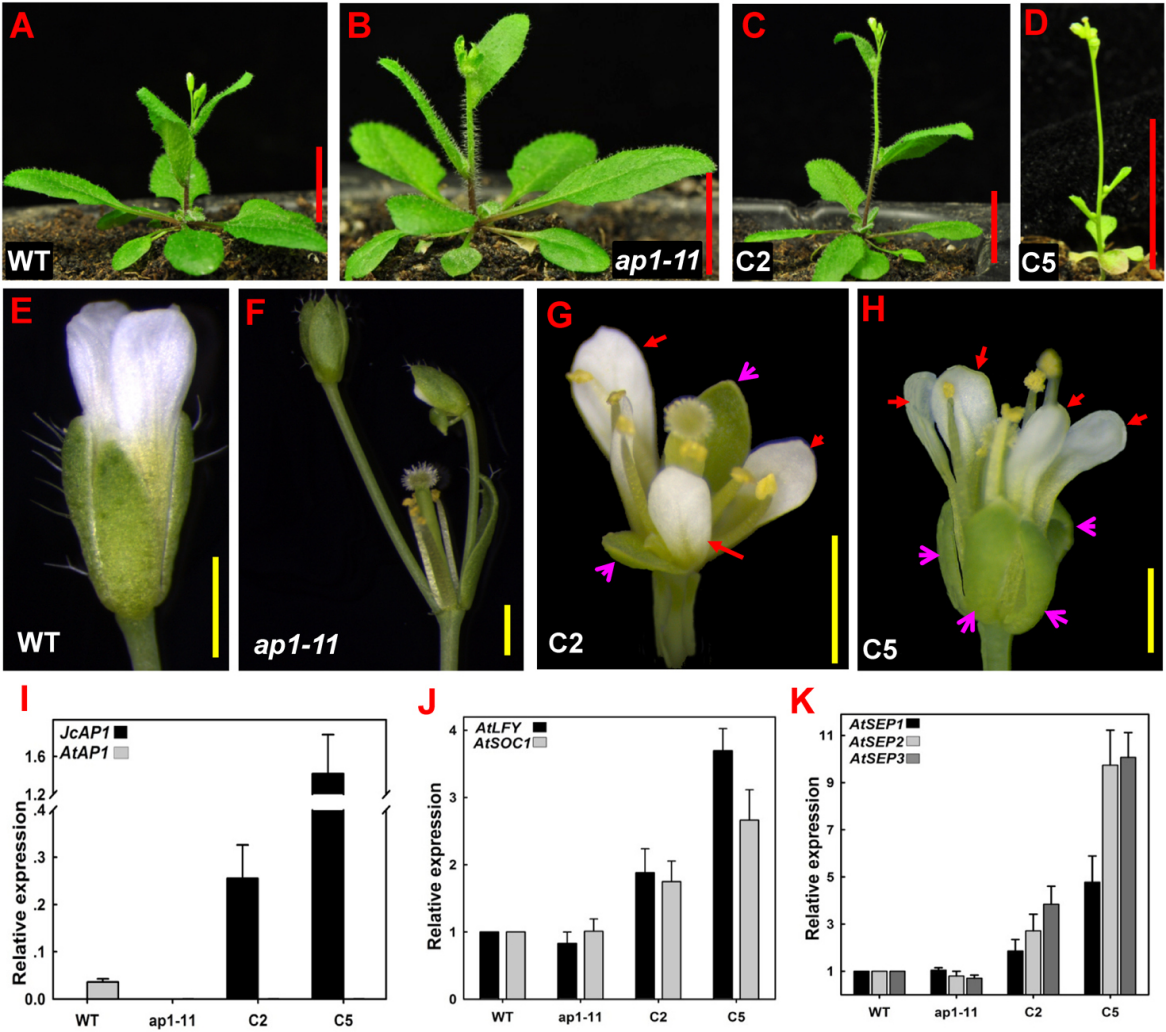
35S:*JcAP1* transgenic Arabidopsis recovers the phenotype of the *ap1-11* mutant and causes early flowering. (A–C) WT, *ap1-11*, and 35S:*JcAP1* complementary *ap1-11* plant line 2 (C2) growth under LD conditions at 30 days after germination. (D) 35S:*JcAP1* complementary *ap1-11* plant line 5 (C5) growth under LD conditions at 15 days after germination. (E–H) Florets of WT (E), *ap1-11* (F), and 35S:*JcAP1* complementary *ap1-11* in which sepals and petals were rescued (G and H). The red arrow indicates that the petals appeared in complementary transgenic plants, and the pink arrow indicates that the sepals appeared in complementary transgenic plants. (I–K) qRT-PCR analysis of *JcAP1* and other flowering-related genes including Arabidopsis *APETALA1* (*AtAP1*), *LEAFY* (*AtLFY*), *SUPPRESSOR OF OVEREXPRESSION OF CONSTANS1* (*AtSOC1*), *SEPALLATA* 1, 2 and 3 (*AtSEP1*, AtSEP2, and *AtSEP3*) in WT, *ap1-11* and transgenic Arabidopsis (C2, C5). The levels of the detected amplicons were normalized using the amplified products of *AtACTIN2*. The mRNA level in WT was set as the standard, with a value of 1. Red bars = 1 cm, yellow bars = 1 mm.

To examine phenotypes, we selected two independent homozygous transgenic lines (C2 and C5) in the T2 generation that showed high *JcAP1* expression levels: line C2 and line C5 (Fig. 4I). Complementary transgenic lines C2 and C5 bolted 6–11 days earlier and produced 2–6 fewer rosette leaves than the controls under LD conditions (Figs. 4A–4D and Table 3). In the extreme complementary transgenic line C5, solitary flowers appeared on the axils of rosette and cauline leaves and terminal flowers appeared on the primary shoots (Fig. 4D). The *ap1-11* mutant flowers lacked petals, and new secondary floral buds developed on the axils of the bract-like organs present in the first whorl (Fig. 4F). The transgenic mutant C2 and C5 lines restored the development of sepals and petals, and axillary flowers were rarely seen at the bracts (Figs. 4G and 4H). Overexpression of *JcAP1* in *ap1* mutant leading to early flowering is because we used a strong promoter, the *35S* promoter, which drives *JcAP1* constitutively expressing. Similar results were found in Arabidopsis *ap1* mutant overexpressing the chrysanthemum and lily *AP1-*like genes (Chen, Lin & Yang, 2008; Shchennikova et al., 2004). Further analysis indicated that the promotion of flowering in the 35S:*JcAP1* transgenic Arabidopsis mutant was correlated with a significant up-regulation of the flower meristem identity genes *AtLFY*, *AtSOC1* and floral organ identity genes *AtSEPs* (Figs. 4J and 4K).

These results demonstrate that the constitutive expression of *JcAP1* complements the defect in floral organ development observed in the *ap1-11* mutant; thus, *JcAP1* functions as an A-class gene in transgenic Arabidopsis.

### Overexpression of *JcAP1* in Jatropha did not cause early flowering

**Figure 5:**
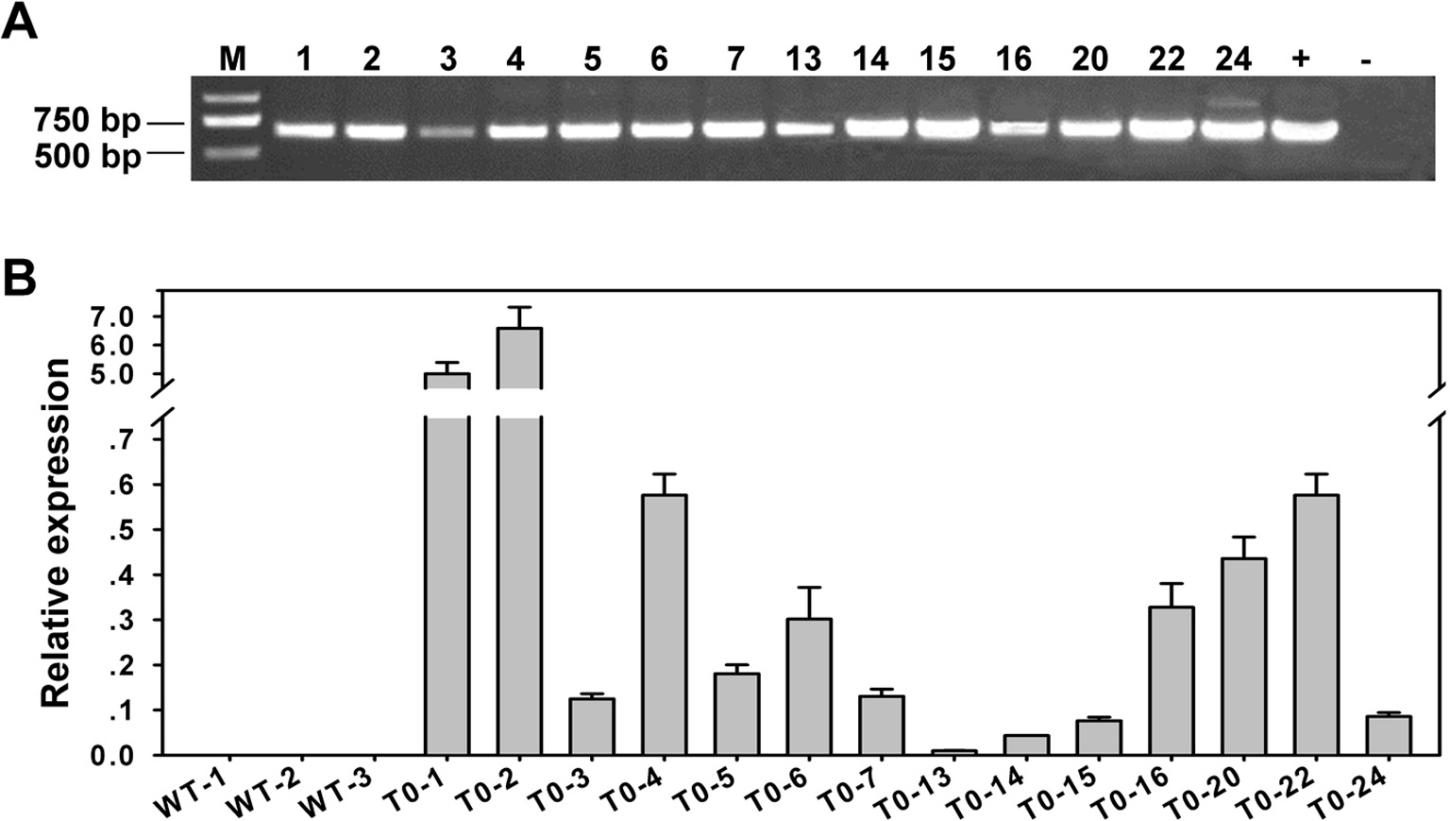
Analysis of the transgenic Jatropha plants. (A) Amplification of the 600-bp fragment containing partial sequences of the 35S promoter and *JcAP1* cDNA. Lanes: M, Trans 2 Kb DNA ladder; +, positive control (plasmid); −, negative control (wild type); and 14 regenerated transgenic Jatropha lines. (B) Quantitative RT-PCR analysis of 3 WT and 14 independent transgenic plants (L1, L2, L3, L4, L5, L6, L7, L13, L14, L15, L16, L20, L22, L24). Two transgenic plants, L2 and L20, showing high and intermediate expression levels, respectively, were chosen for further analysis. RNA was extracted from young leaves of 2-month-old plantlets. The transcript levels were normalized using the *JcACTIN1* gene as a reference.

**Figure 6:**
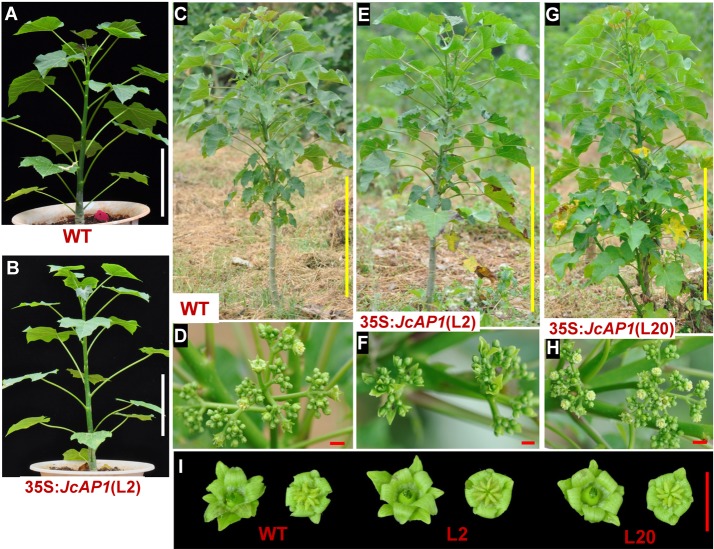
The flowering time of 35S:*JcAP1* transgenic Jatropha in the field. (A) WT plant grown in a pot for 2 months, at the vegetative growth stage; (B) 35S:*JcAP1* transgenic Jatropha grown in a pot for 2 months, at the vegetative growth stage. (C) WT plants grown in the field for 4 months, at the anthesis stage; (D) The inflorescence of WT Jatropha in the field. (E, G) 35S:*JcAP1* transgenic Jatropha L2 and L20 plants grown in the field for 4 months, at the anthesis stage; (F, H) The inflorescence of transgenic Jatropha in the field. (I) The flowers of WT and 35S:*JcAP1* transgenic Jatropha L2 and L20 plants. Red bars = 1 cm, white bars = 10 cm, yellow bars = 50 cm.

Transgenic analysis performed in Arabidopsis suggested that *JcAP1* might act as a floral identity gene in Jatropha. To test this hypothesis, we generated transgenic Jatropha with the 35S:*JcAP1* construct (Fig. 3A) as previously described (Pan, Fu & Xu, 2010). Non-transgenic plants were used as a control. Fifty-five independent transgenic lines were confirmed via PCR using genomic DNA isolated from leaves of 2-month-old plantlets. And the partial results are shown in Fig. 5A. Next, *JcAP1* expression levels in fourteen PCR-positive lines were examined through qRT-PCR using RNA extracted from young leaves of 2-month-old plantlets (Fig. 5B). To our surprise, all of these transgenic Jatropha lacked an early-flowering phenotype (Figs. 6A and 6B). When regenerated plantlets were grown in the field for 4 months, flower buds emerged in both transgenic and control plants (Figs. 6C–6H). We chose L2 and L20, which exhibited high and intermediate expression levels, respectively (Fig. 5B), to further analyze the expression levels of several floral identity-related genes in the shoot apices of 6-month-old plantlets. The results showed that the transcript levels of *JcLFY*, *JcSOC1* and *JcTFL1s* (Fig. S2) were not significantly altered in both transgenic lines. The 35S promoter was highly active in the Jatropha inflorescence buds (Tao et al., 2015), but the inflorescence structure (Figs. 6D, 6F and 6H) and floral organ pattern (Fig. 6I) were not obviously different. These results indicate that *JcAP1* may be inadequate to promote flowering and floral organ development by itself in Jatropha.

## Discussion

Recently, Jatropha has garnered significant attention as a potential oilseed plant for the production of renewable biofuel. Despite the interest in this woody oil plant, relatively little is known regarding the molecular biology of this species compared with more established oilseed crops, such as rapeseed (Handa, 2003; Kresovich et al., 1995) and castor bean (Chan et al., 2010).

Amino acid sequence similarity, protein structures, and phylogenetic analysis suggested that JcAP1 exhibits a similar function to other AP1 homologues. In Arabidopsis, *AP1* functions redundantly with *FUL* in specifying floral meristem identity (Bowman et al., 1993). However, in the phylogenetic tree, AP1 and FUL clustered in the separate clades indicated the functional divergence between the two paralogs. Except for floral meristem determination, *FUL* is required for proper fruit and leaf development in Arabidopsis (Gu et al., 1998), and *FUL* prevents normal senescence and winter dormancy in woody *Populus tremula* L (Hoenicka et al., 2008). JcAP1 was clustered in the AP1 clade suggesting this gene may functions similarly as other AP1 genes in the regulation of flowering and flower organ development.

Quantitative RT-PCR results showed that *JcAP1* transcripts were highly expressed in inflorescence buds, flower buds, sepals and petals (Fig. 2). This expression pattern is consistent with that of *AtAP1* in Arabidopsis (Mandel et al., 1992). The expression profiles revealed that the highest *JcAP1* transcript levels occurred in the earlier stages of male and female flower buds (Fig. 2A), implying that *JcAP1* may play a role in maintaining the normal development of flower patterns (Collaudin, 2012). In addition, the high *JcAP1* expression levels observed in sepals and petals (Fig. 2B) suggested that *JcAP1* may be involved in sepal and petal development. In Arabidopsis, the function of *AP1* in the regulation of sepal and petal development was revealed using an *ap1* mutant (Mandel et al., 1992). The *AP1* gene was identified as a member of the floral meristem identity genes, which largely acted downstream of the floral integrators *FT*, *LFY*, and *SOC1* (Bowman et al., 1993; Liljegren et al., 1999; Liu et al., 2013; Riechmann, Krizek & Meyerowitz, 1996). In this study, we showed that overexpression of *JcAP1* in Arabidopsis resulted in reduced vegetative growth, early flowering and the formation of terminal and solitary flowers (Fig. 3; Tables 1, and 2). These findings are similar to the phenotypic changes caused by constitutive expression of *AP1* homologs in Arabidopsis (Chi et al., 2011; Kotoda et al., 2002; Liljegren et al., 1999; Liu et al., 2013; Mandel & Yanofsky, 1995; Sun et al., 2014; Wang et al., 2013; Weigel & Nilsson, 1995; Winterhagen et al., 2013). The production of terminal and solitary flowers in *AP1-* overexpressing plants is due to the inhibition of *TFL1* expression induced by *AP1* (Blazquez et al., 2006). In Arabidopsis, young seedlings showed weak *TFL1* expression; the *TFL1* expression increased after 8 days and young inflorescences showed the strongest *TFL1* expression (Bradley et al., 1997). In previous research, *TFL1* down-regulated directly by *AP1* was confirmed by the CHIP-Seq analysis (Kaufmann et al., 2010). Compared with WT plants, the *TFL1* expression was not detected in primary shoot apices and secondary meristems in *AP1* overexpressing plants (Liljegren et al., 1999). Similarly, in our research, when *JcAP1*was overexpressed in Arabidopsis, the expression of *TFL1* was also suppressed. The aboveground tissues of 15-day-old plants were used for detecting the *TFL1* expression in our study. The inflorescence buds had appeared in *JcAP1* overexpressing plants while WT plants were still in vegetative growth. According to the *TFL1* expression pattern in Arabidopsis, in which the inflorescences showed the highest expression level, *TFL1* expression in transgenic plant was supposed to be higher than in WT plants. However, the expression level of *TFL1* was decreased in the *JcAP1* highly expressing line (L12) (Fig. S1L). This result indicated that the increase of *JcAP1* expression repressed the *TFL1* expression. Consistently, when *JcAP1* was lowly expressed (L30), the expression level of *TFL1* was markedly increased (Fig. S1L). In Jatropha, nevertheless, the expression of three *JcTFL1s* (*JcTFL1a*, *JcTFL1b* and *JcTFL1c*) were not significantly altered in *JcAP1* overexpressing plants (Fig. S2D). Thus, we supposed that *JcTFL1* was indirectly relative to *JcAP1*.

Overexpression of *JcAP1* in the Arabidopsis *ap1-11* mutant resulted in early flowering, restoration of sepal and petal development, and repression of secondary flower formation in the bract axils (Figs. 5I–5K). These results are consistent with the phenotypic changes observed following the ectopic expression of Chrysanthemum and lily *AP1*-like genes in the Arabidopsis *ap1* mutant (Chen, Lin & Yang, 2008; Shchennikova et al., 2004). These findings imply that *JcAP1* acts as a functional homolog of *AP1* in Arabidopsis.

In contrast to *JcAP1*-overexpressing Arabidopsis, *JcAP1*-overexpressing Jatropha did not exhibit early flowering (Fig. 6). But the expression profile showed that *JcAP1* was predominantly expressed in inflorescence buds and flower buds (Fig. 2A). It indicates that *JcAP1* may be insufficient to regulate flowering time or floral organ development by itself in Jatropha. Similar to our findings, overexpression of the Populus ortholog of *APETALA1* in Arabidopsis led to early flowering whereas it failed to promote flowering in hybrid aspen (Azeez et al., 2014). In addition new functions of *AP1* homologs have been identified in several species. The *AP1* homolog of grapevine (*VAP1*) is involved in the formation of tendrils (Calonje et al., 2004). Wheat *AP1* (*WAP1*) has no known role in flower development but is required for vernalization and phase transition (Danyluk et al., 2003; Handa, 2003; Trevaskis et al., 2003). In tomato plants, *AP1/FUL* MADS box genes are involved in tomato leaf development (Burko et al., 2013).

The molecular mechanisms controlling flowering in perennials have not been studied as extensively as those of annual plants (Albani & Coupland, 2010). It seems more complex in perennial plants in that a well-known identified flowering gene is unable to control the flowering time. For example, overexpression of *LFY* failed to cause early flowering in apple (Flachowsky et al., 2010) and hybrid populous (Rottmann et al., 2000). Overexpression of miR172, which caused extremely early flowering in Arabidopsis (Aukerman & Sakai, 2003), was unable to promote flowering in the perennial plant *Cardamine flexuosa* unless it was treated with vernalization simultaneously (Zhou et al., 2013). In this study, the results also suggest that *JcAP1* itself may not be sufficient to promote flowering in Jatropha; perhaps it needs to be associated with other factors to function in these processes. Other important floral identity genes or environment factors need to be characterized in Jatropha in the future study.

## Supplemental Information

10.7717/peerj.1969/supp-1 Table S1 Primers used for all experiments

10.7717/peerj.1969/supp-2 Figure S1 Quantitative RT-PCR analysis of *JcAP1* and other flower-related genes in WT and transgenic Arabidopsis (A) The expression level of JcAP1 in WT and transgenic Arabidopsis L12 L30 plants; JcAP1 expression was not detected in WT; transcript levels were not normalized. (B–L) The expression levels of *AtLFY*, *AtAP1*, *AtFUL*, AtAG, *AtAP3*, *AtSEP1*, *AtSEP2*, AtSEP3, *AtFT*, AtCAL, *and AtTFL1*, respectively. RNA sample extracted from apex and rosette leaves of 35S: *JcAP1* transgenic and WT plants cultured for 15 days in a pot. Transcript levels were normalized using the *AtACTIN2* gene as a reference. The mRNA level in WT was set as the standard, with a value of 1.

10.7717/peerj.1969/supp-3 Figure S2 Quantitative RT-PCR analysis of *JcAP1* and flower-related genes in WT and 35S:*JcAP1* transgenic Jatropha The expression levels of *JcAP1*, *JcLFY*, *JcSOC1*, and *JcTFL1s* were detected in shoot apices of 6-month-old plantlets of WT and transgenic Jatropha. The qRT-PCR results were obtained using two independent biological replicates and three technical replicates for each RNA sample extracted from the apex of the 35S: *JcAP1* transgenic and WT shoots. Transcript levels were normalized using the *JcACTIN1* gene as a reference. The mRNA level in WT was set as the standard, with a value of 1.

10.7717/peerj.1969/supp-4 Figure S3 Flower morphological characteristics of Jatropha in different developmental stages (A) Inflorescence bud stage 1 (IB1): 0–5 days, inflorescence buds are visible; (B) inflorescence bud stage 2 (IB2): 1 week after IB1; (C) inflorescence bud stage 3 (IB3): 1 week after IB2; (D) flower bud stage 1 (FB1): 1 week after IB3; (E) flower bud stage 2 (FB2): male flower buds (MFB) and female flower buds (FFB) are identifiable one week after FB1; (F): male and female flower stage: male flowers (MF) and female flowers (FF) bloomed one week after FB2. In (E) and (F), red arrows indicate FFBs and FFs, respectively; and pink arrows indicate MFBs and MFs, respectively. Bars = 1 cm.

10.7717/peerj.1969/supp-5 Data S1 Raw data Transgenic arabidopsis flowering time and gene expression levels.

## Abbreviations

AG: AGAMOUS
AP1: APETALA 1
AP3: APETALA 3
CAL: CAULIFLOWER
CaMV: Cauliflower Mosaic Virus
FT: FLOWERING LOCUS T
FUL: FRUITFULL
LD: long day
LFY: LEAFY
qRT-PCR: Quantitative reverse transcriptase-polymerase chain reaction
SD: short day
SEP: SEPALLATA
SOC1: SUPPRESSOR OF OVEREXPRESSION OF CONSTANS1
TFL1: TERMINAL FLOWER 1

## Additional Information and Declarations

### DNA Deposition

The following information was supplied regarding the deposition of DNA sequences: GenBank accession no. KR013222; GenBank accession no. KU942379.

### Data Availability

The following information was supplied regarding data availability: The raw data has been supplied as Data S1.

## References

[ref-1] Akashi K (2012). *Jatropha* research: a new frontier for biofuel development. Plant Biotechnology.

[ref-2] Albani MC, Coupland G, Marja CPT (2010). Comparative analysis of flowering in annual and perennial plants. Current topics in developmental biology.

[ref-3] Alvarez-Buylla ER, Garcia-Ponce B, Garay-Arroyo A (2006). Unique and redundant functional domains of *APETALA1* and *CAULIFLOWER*, two recently duplicated *Arabidopsis thaliana* floral MADS-box genes. Journal of Experimental Botany.

[ref-4] Aukerman MJ, Sakai H (2003). Regulation of flowering time and floral organ identity by a microRNA and its APETALA2-like target genes. Plant Cell.

[ref-5] Azeez A, Miskolczi P, Tylewicz S, Bhalerao R (2014). A tree ortholog of *APETALA1* mediates photoperiodic control of seasonal growth. Current Biology.

[ref-6] Blazquez MA, Ferrandiz C, Madueno F, Parcy F (2006). How floral meristems are built. Plant Molecular Biology.

[ref-7] Bowman JL, Alvarez J, Weigel D, Meyerowitz EM, Smyth DR (1993). Control of flower development in *Arabidopsis thaliana* by *APETALA 1* and interacting genes. Development.

[ref-8] Bradley D, Ratcliffe O, Vincent C, Carpenter R, Coen E (1997). Inflorescence commitment and architecture in *Arabidopsis*. Science.

[ref-9] Burko Y, Shleizer-Burko S, Yanai O, Shwartz I, Zelnik ID, Jacob-Hirsch J, Kela I, Eshed-Williams L, Ori N (2013). A role for APETALA1/FRUITFULL transcription factors in tomato leaf development. Plant Cell.

[ref-10] Calonje M, Cubas P, Martínez-Zapater JM, Carmona MJ (2004). Floral meristem identity genes are expressed during tendril development in *grapevine*. Plant Physiology.

[ref-11] Chan AP, Crabtree J, Zhao Q, Lorenzi H, Orvis J, Puiu D, Melake-Berhan A, Jones KM, Redman J, Chen G (2010). Draft genome sequence of the oilseed species *Ricinus communis*. Nature Biotechnology.

[ref-12] Chen A, Li C, Hu W, Lau MY, Lin H, Rockwell NC, Martin SS, Jernstedt JA, Lagarias JC, Dubcovsky J (2014). PHYTOCHROME C plays a major role in the acceleration of wheat flowering under long-day photoperiod. Proceedings of the National Academy of Sciences of the United States of America.

[ref-13] Chen M-K, Lin I-C, Yang C-H (2008). Functional analysis of three lily (*Lilium longiflorum*) APETALA1-like MADS box genes in regulating floral transition and formation. Plant Cell Physiology.

[ref-14] Chi Y, Huang F, Liu H, Yang S, Yu D (2011). An *APETALA1-*like gene of soybean regulates flowering time and specifies floral organs. Journal of Plant Physiology.

[ref-15] Clough SJ, Bent AF (1998). Floral dip: a simplified method for *Agrobacterium*—mediated transformation of *Arabidopsis thaliana*. Plant Journal.

[ref-16] Collaudin S (2012). Morphogenesis of the flower of Arabidopsis, genes networks and mathematical modelling. Organ.

[ref-17] Danyluk J, Kane NA, Breton G, Limin AE, Fowler DB, Sarhan F (2003). TaVRT-1, a putative transcription factor associated with vegetative to reproductive transition in cereals. Plant Physiology.

[ref-18] De Oliveira RR, Cesarino I, Mazzafera P, Dornelas MC (2014). Flower development in *Coffea arabica* L.: new insights into MADS-box genes. Plant Reproduction.

[ref-19] Ding L-W, Sun Q-Y, Wang Z-Y, Sun Y-B, Xu Z-F (2008). Using silica particles to isolate total RNA from plant tissues recalcitrant to extraction in guanidine thiocyanate. Analytical Biochemistry.

[ref-20] Divakara B, Upadhyaya H, Wani S, Gowda C (2010). Biology and genetic improvement of *Jatropha curcas* L.: a review. Applied Energy.

[ref-21] Ferrándiz C, Gu Q, Martienssen R, Yanofsky MF (2000). Redundant regulation of meristem identity and plant architecture by *FRUITFULL*, *APETALA1* and *CAULIFLOWER*. Development.

[ref-22] Flachowsky H, Haettasch C, Hoefer M, Peil A, Hanke M-V (2010). Overexpression of *LEAFY* in apple leads to a columnar phenotype with shorter internodes. Planta.

[ref-23] Fu Q, Li C, Tang M, Tao Y-B, Pan B-Z, Zhang L, Niu L, He H, Wang X, Xu Z-F (2015). An efficient protocol for *Agrobacterium*-mediated transformation of the biofuel plant *Jatropha curcas* by optimizing kanamycin concentration and duration of delayed selection. Plant Biotechnology Reports.

[ref-24] Ghosh A, Chaudhary DR, Reddy MP, Rao SN, Chikara J, Pandya JB, Patolia JS, Gandhi MR, Adimurthy S, Vaghela N, Mishra S, Rathod MR, Prakash AR, Shethia BD, Upadhyay SC, Balakrishna V, Prakash R, Ghosh PK (2007). Prospects for Jatropha methyl ester (biodiesel) in India. International Journal of Environmental Studies.

[ref-25] Ghosh A, Chikara J, Chaudhary DR, Prakash A, Boricha G, Zala A (2010). Paclobutrazol arrests vegetative growth and unveils unexpressed yield potential of *Jatropha curcas*. Journal of Plant Growth Regulation.

[ref-26] Gu Q, Ferrandiz C, Yanofsky MF, Martienssen R (1998). The *FRUITFULL MADS*-box gene mediates cell differentiation during *Arabidopsis* fruit development. Development.

[ref-27] Gu K, Tian D, Mao H, Wu L, Yin Z (2015). Development of marker-free transgenic *Jatropha curcas* producing curcin-deficient seeds through endosperm-specific RNAi-mediated gene silencing. BMC Plant Biology.

[ref-28] Han Y, Zhang C, Yang H, Jiao Y (2014). Cytokinin pathway mediates *APETALA1* function in the establishment of determinate floral meristems in *Arabidopsis*. Proceedings of the National Academy of Sciences of the United States of America.

[ref-29] Handa H (2003). The complete nucleotide sequence and RNA editing content of the mitochondrial genome of rapeseed (*Brassica napus* L.): comparative analysis of the mitochondrial genomes of rapeseed and *A rabidopsis thaliana*. Nucleic Acids Research.

[ref-30] Hirakawa H, Tsuchimoto S, Sakai H, Nakayama S, Fujishiro T, Kishida Y, Kohara M, Watanabe A, Yamada M, Aizu T (2012). Upgraded genomic information of *Jatropha curcas* L. Plant Biotechnology.

[ref-31] Hoenicka H, Nowitzki O, Hanelt D, Fladung M (2008). Heterologous overexpression of the birch *FRUITFULL*-like MADS-box gene *BpMADS4* prevents normal senescence and winter dormancy in Populus tremula L. Planta.

[ref-32] Irish VF, Sussex IM (1990). Function of the apetala-1 gene during *Arabidopsis* floral development. Plant Cell.

[ref-33] Kajikawa M, Morikawa K, Inoue M, Widyastuti U, Suharsono S, Yokota A, Akashi K (2012). Establishment of bispyribac selection protocols for *Agrobacterium tumefaciens*-and *Agrobacterium rhizogenes*-mediated transformation of the oil seed plant *Jatropha curcas* L. Plant Biotechnology.

[ref-34] Kaufmann K, Wellmer F, Muino JM, Ferrier T, Wuest SE, Kumar V, Serrano-Mislata A, Madueno F, Krajewski P, Meyerowitz EM, Angenent GC, Riechmann JL (2010). Orchestration of floral initiation by *APETALA1*. Science.

[ref-35] Khalil HPSA, Aprilia NAS, Bhat AH, Jawaid M, Paridah MT, Rudi D (2013). A *Jatropha* biomass as renewable materials for biocomposites and its applications. Renewable & Sustainable Energy Reviews.

[ref-36] King AJ, Montes LR, Clarke JG, Itzep J, Perez CA, Jongschaap RE, Visser RG, Van Loo EN, Graham IA (2015). Identification of QTL markers contributing to plant growth, oil yield and fatty acid composition in the oilseed crop *Jatropha curcas* L. Biotechnology for Biofuels.

[ref-37] Kotoda N, Wada M, Kusaba S, Kano-Murakami Y, Masuda T, Soejima J (2002). Overexpression of *MdMADS5*, an *APETALA1*-like gene of apple, causes early flowering in transgenic *Arabidopsis*. Plant Science.

[ref-38] Kresovich S, Szewc-McFadden A, Bliek S, McFerson J (1995). Abundance and characterization of simple-sequence repeats (SSRs) isolated from a size-fractionated genomic library of *Brassica napus* L. (rapeseed). Theoretical and Applied Genetics.

[ref-39] Krogan NT, Ashton NW (2000). Ancestry of plant MADS-box genes revealed by bryophyte (Physcomitrella patens) homologues. New Phytologist.

[ref-40] Kumar N, Anand KGV, Pamidimarri DVNS, Sarkar T, Reddy MP, Radhakrishnan T, Kaul T, Reddy MK, Sopori SK (2010). Stable genetic transformation of *Jatropha curcas* via Agrobacterium tumefaciens-mediated gene transfer using leaf explants. Industrial Crops and Products.

[ref-41] Li C, Luo L, Fu Q, Niu L, Xu Z-F (2014). Isolation and functional characterization of *JcFT*, a *FLOWERING LOCUS T (FT)* homologous gene from the biofuel plant *Jatropha curcas*. BMC Plant Biology.

[ref-42] Liljegren SJ, Gustafson-Brown C, Pinyopich A, Ditta GS, Yanofsky MF (1999). Interactions among *APETALA1, LEAFY*, and *TERMINAL FLOWER1* specify meristem fate. Plant Cell.

[ref-43] Litt A, Irish VF (2003). Duplication and diversification in the *APETALA1/FRUITFULL* floral homeotic gene lineage: implications for the evolution of floral development. Genetics.

[ref-44] Liu L, Zhu Y, Shen L, Yu H (2013). Emerging insights into florigen transport. Current Opinion in Plant Biology.

[ref-45] Livak KJ, Schmittgen TD (2001). Analysis of relative gene expression data using real-time quantitative PCR and the 2 }{}${}^{\Delta \Delta \mathrm{CT}}$ method. Methods.

[ref-46] Mandel MA, Gustafson-Brown C, Savidge B, Yanofskay MF (1992). Molecular characterization of the *Arabidopsis* floral homeotic gene *APETALA1*. Nature.

[ref-47] Mandel MA, Yanofsky MF (1995). A gene triggering flower formation in *Arabidopsis*. Nature.

[ref-48] Mao HZ, Ye J, Chua NH (2013). Genetic transformation of *Jatropha curcas*.

[ref-49] Misra P, Toppo DD, Mishra MK, Saema S, Singh G (2012). *Agrobacterium tumefaciens*-mediated transformation protocol of *Jatropha curcas* L. using leaf and hypocotyl segments. Journal of Plant Biochemistry and Biotechnology.

[ref-50] Mofijur M, Rasul M, Hyde J, Azad A, Mamat R, Bhuiya M (2016). Role of biofuel and their binary (diesel–biodiesel) and ternary (ethanol–biodiesel–diesel) blends on internal combustion engines emission reduction. Renewable & Sustainable Energy Reviews.

[ref-51] Ng M, Yanofsky MF (2001). Activation of the *Arabidopsis* B class homeotic genes by *APETALA1*. Plant Cell.

[ref-52] Ong H, Mahlia T, Masjuki H, Norhasyima R (2011). Comparison of *palm* oil, *Jatropha curcas* and *Calophyllum inophyllum* for biodiesel: a review. Renewable & Sustainable Energy Reviews.

[ref-53] Pan J, Fu Q, Xu Z-F (2010). *Agrobacterium tumefaciens*-mediated transformation of biofuel plant *Jatropha curcas* using kanamycin selection. African Journal of Biotechnology.

[ref-54] Pan B-Z, Xu Z-F (2011). Benzyladenine treatment significantly increases the seed yield of the biofuel plant *Jatropha curcas*. Journal of Plant Growth Regulation.

[ref-55] Pandey VC, Singh K, Singh JS, Kumar A, Singh B, Singh RP (2012). *Jatropha curca*s: a potential biofuel plant for sustainable environmental development. Renewable & Sustainable Energy Reviews.

[ref-56] Peña L, Martín-Trillo M, Juárez J, Pina JA, Navarro L, Martínez-Zapater JM (2001). Constitutive expression of *Arabidopsis LEAFY* or *APETALA1* genes in citrus reduces their generation time. Nature Biotechnology.

[ref-57] Pramanik K (2003). Properties and use of *Jatropha curcas* oil and diesel fuel blends in compression ignition engine. Renewable Energy.

[ref-58] Pua F-l, Fang Z, Zakaria S, Guo F, Chia C-H (2011). Direct production of biodiesel from high-acid value *Jatropha* oil with solid acid catalyst derived from lignin. Biotechnology for Biofuels.

[ref-59] Riechmann JL, Krizek BA, Meyerowitz EM (1996). Dimerization specificity of *Arabidopsis MADS* domain homeotic proteins *APETALA1, APETALA3, PISTILLATA*, and *AGAMOUS*. Proceedings of the National Academy of Sciences of the United States of America.

[ref-60] Rottmann WH, Meilan R, Sheppard LA, Brunner AM, Skinner JS, Ma CP, Cheng SP, Jouanin L, Pilate G, Strauss SH (2000). Diverse effects of overexpression of *LEAFY* and *PTLF*, a poplar (Populus) homolog of *LEAFY/FLORICAULA*, in transgenic poplar and Arabidopsis. Plant Journal.

[ref-61] Sato S, Hirakawa H, Isobe S, Fukai E, Watanabe A, Kato M, Kawashima K, Minami C, Muraki A, Nakazaki N, Takahashi C, Nakayama S, Kishida Y, Kohara M, Yamada M, Tsuruoka H, Sasamoto S, Tabata S, Aizu T, Toyoda A, Shin-i T, Minakuchi Y, Kohara Y, Fujiyama A, Tsuchimoto S, Kajiyama SI, Makigano E, Ohmido N, Shibagaki N, Cartagena JA, Wada N, Kohinata T, Atefeh A, Yuasa S, Matsunaga S, Fukui K (2011). Sequence analysis of the genome of an oil-bearing tree, *Jatropha curcas* L. DNA Research.

[ref-62] Shchennikova AV, Shulga OA, Immink R, Skryabin KG, Angenent GC (2004). Identification and characterization of four chrysanthemum MADS-box genes, belonging to the APETALA1/FRUITFULL and SEPALLATA3 subfamilies. Plant Physiology.

[ref-63] Sinha P, Islam MA, Negi MS, Tripathi SB (2015). Changes in oil content and fatty acid composition in *Jatropha curcas* during seed development. Industrial Crops and Products.

[ref-64] Sun L-M, Zhang J-Z, Mei L, Hu C-G (2014). Molecular cloning, promoter analysis and functional characterization of *APETALA 1*-like gene from precocious trifoliate orange (*Poncirus trifoliata*). Scientia Horticulturae.

[ref-65] Tao Y-B, He L-L, Niu L-J, Xu Z-F (2015). Isolation and characterization of an ubiquitin extension protein gene (JcUEP) promoter from *Jatropha curcas*. Planta.

[ref-66] Tjeuw J, Slingerland M, Giller K (2015). Relationships among *Jatropha curca*s seed yield and vegetative plant components under different management and cropping systems in Indonesia. Biomass & Bioenergy.

[ref-67] Trevaskis B, Bagnall DJ, Ellis MH, Peacock WJ, Dennis ES (2003). MADS box genes control vernalization-induced flowering in cereals. Proceedings of the National Academy of Sciences of the United States of America.

[ref-68] Tuskan GA, Difazio S, Jansson S, Bohlmann J, Grigoriev I, Hellsten U, Putnam N, Ralph S, Rombauts S, Salamov A (2006). The genome of black cottonwood, *Populus trichocarpa* (Torr. & Gray). Science.

[ref-69] Wagner D, Sablowski RWM, Meyerowitz EM (1999). Transcriptional activation of *APETALA1* by *LEAFY*. Science.

[ref-70] Wang J, Zhang X, Yan G, Zhou Y, Zhang K (2013). Over-expression of the *PaAP1* gene from sweet cherry (*Prunus avium L*.) causes early flowering in *Arabidopsis thaliana*. Journal of Plant Physiology.

[ref-71] Weigel D, Nilsson O (1995). A developmental switch sufficient for flower initiation in diverse plants. Nature.

[ref-72] William DA, Su YH, Smith MR, Lu M, Baldwin DA, Wagner D (2004). Genomic identification of direct target genes of LEAFY. Proceedings of the National Academy of Sciences of the United States of America.

[ref-73] Winterhagen P, Tiyayon P, Samach A, Hegele M, Wünsche J (2013). Isolation and characterization of *FLOWERING LOCUS T* subforms and *APETALA1* of the subtropical fruit tree *Dimocarpus longan*. Plant Physiology and Biochemistry.

[ref-74] Wu J, Liu Y, Tang L, Zhang F, Chen F (2011). A study on structural features in early flower development of *Jatropha curcas L.* and the classification of its inflorescences. African Journal of Agricultural Research.

[ref-75] Wu PZ, Zhou CP, Cheng SF, Wu ZY, Lu WJ, Han JL, Chen YB, Chen Y, Ni PX, Wang Y, Xu X, Huang Y, Song C, Wang ZW, Shi N, Zhang XD, Fang XH, Yang Q, Jiang HW, Chen YP, Li MR, Wang Y, Chen F, Wang J, Wu GJ (2015). Integrated genome sequence and linkage map of physic nut (*Jatropha curcas* L.), a biodiesel plant. Plant Journal.

[ref-76] Zhou C-M, Zhang T-Q, Wang X, Yu S, Lan H, Tang H, Feng Z-Y, Zozomova-Lihova J, Wang J-W (2013). Molecular basis of age-dependent vernalization in cardamine flexuosa. Science.

