# Supplementary material for: Ectopic expression of Jatropha curcas APETALA1 (JcAP1) caused early flowering in Arabidopsis, but not in Jatropha
Source: PeerJ. 2016 Apr 25;4:e1969. doi: 10.7717/peerj.1969 (PMC4860315; doi:10.7717/peerj.1969)
Supplement: Table S1 [file peerj-04-1969-s001.docx]

**Table S1 Primers used for all experiments**

| **Target template** | **Primer name** | **Primer sequence** |
| --- | --- | --- |
| *JcAP1* full length | XK928-KpnI F | GGGGTACCCCGGAAAGAAGAGGAAAAATTTATACA |
|  | XK929-SalI R | GCGTCGACGTAGCATGTAGTAATCTCTCTCCTGTT |
| *JcAP1* qRT-PCR | XA311 F | TAACAGACTCAAGGCGAAGGT |
|  | XA312 R | AGTTGGTTGTTTCTTGCTCGG |
| *JcLFY* qRT-PCR | XT655 F | GGATAAGATACTACACAGCAGCGA |
|  | XT656 R | TAACCCTTCTTGAGAGAGAGCATC |
| *JcSOC1* qRT-PCR | XK656 F | TTCTTGGACGGCAACGCTTA |
|  | XK657 R | CTCTCGGAAAAGTGTGGGATC |
| *JcTFL1a* qRT-PCR | XA520 F | GTGTATGTTAGTACCGTATTTGGAT |
|  | XA521 R | CTAAACCAAAGAGCTTATTCTAGGC |
| *JcTFL1b* qRT-PCR | XA485 F | ACCAGTAGACCCTCTTATTGTTGAGA |
|  | XA486 R | TCATATCATCTCCTTCCACAGCAACT |
| *JcTFL1c* qRT-PCR | XA203 F | ACGGAGCCACAGCCACTTACTGTAG |
|  | XA204 R | ACTCTAGGTTTAGCAGCAATGACCG |
| *AtAP1* qRT-PCR | XT803 F | TTTGGAGAGAAACCAGAGGCATT |
|  | XT804 R | GTAAGGATGCTGGATTTGGTGCT |
| *AtLFY* qRT-PCR | XT805 F | TGCTCTCTCCCAAGAAGGGTTAT |
|  | XT806 R | TTGGTTTCTTTCTCCGTCTCTGC |
| *AtAP3* qRT-PCR | XT896 F | GTCTTGAGGATGAAATGGAAAAC |
|  | XT897 R | TGGTATCCAAGAACTGAGTCGTA |
| *AtAG* qRT-PCR | XT898 F | GCGTCAACAAATAATCAGCATAC |
|  | XT899 R | CGAAGAATCTGGTTATCGTTATG |
| *AtFLC* qRT-PCR | XT900 F | CTTTCTGTTCTCTGTGACGCATC |
|  | XT901 R | AGTCTCAAGGTGTTCCTCCAGTT |
| *AtFUL* qRT-PCR | XT902 F | GCTATCAAGAGCATTAGGTCAAG |
|  | XT903 R | GTTACGCAGTATTGAGGCAGAA |
| *AtCAL* qRT-PCR | XT904 F | GGAGAGAAACCAAAGGCATTATC |
|  | XT905 R | TCCTTTCTTTGGAGGTGGTTGA |
| *AtTFL1* qRT-PCR | XT906 F | ATAATGGGGAGAGTGGTAGGAGA |
|  | XT907 R | TCTGGGTCTATCATCACCAAAGT |
| *AtSEP1* qRT-PCR | XK904 F | CTTCTTGGGGAGGATTTAGGA |
|  | XK905 R | ACATTCTGTTCACCACCTTCC |
| *AtSEP2* qRT-PCR | XK906 F | GGACATCCTCAGGCTCATTCTC |
|  | XK907 R | AGAAGTATCGCTCACAGCATCC |
| *AtSEP3* qRT-PCR | XK908 F | AATGGGAAGAGGGAGAGTAGA |
|  | XK909 R | TTCTGGTGCTCCATAGTTACA |
| *AtFT* qRT-PCR | XA358 F | AGAGGTGACTAATGGCTTGGAT |
|  | XA359 R | AAGGTTGTTCCAGTTGTAGCAG |
| *JcACTIN1* qRT-PCR | XK191 F | CTCCTCTCAACCCCAAAGCCAA |
|  | XK192 R | CACCAGAATCCAGCACGATACCA |
| *AtACTIN2* qRT-PCR | XK718 F | TGTGCCAATCTACGAGGGTTT |
|  | XK719 R | TTTCCCGCTCTGCTGTTGT |

**Figure S1 Quantitative RT-PCR analysis of *JcAP1* and other flower-related genes in WT and transgenic Arabidopsis.** (A) The expression level of *JcAP1* in WT and transgenic Arabidopsis L12 L30 plants; *JcAP1* expression was not detected in WT; transcript levels were not normalized. (B-L) The expression levels of *AtLFY*, *AtAP1*, *AtFUL*, *AtAG*, *AtAP3*, *AtSEP1*, *AtSEP2*, *AtSEP3*, *AtFT*, *AtCAL,* and *AtTFL1*, respectively*.* RNA sample extracted from apex and rosette leaves of 35S:*JcAP1* transgenic and WT plants cultured for 15 days in a pot. Transcript levels were normalized using the *AtACTIN2* gene as a reference. The mRNA level in WT was set as the standard, with a value of 1.

**Figure S2 Quantitative RT-PCR analysis of *JcAP1* and flower-related genes in WT and 35S:*JcAP1* transgenic Jatropha.** The expression levels of *JcAP1*, *JcLFY*, *JcSOC1*, and *JcTFL1s* were detected in shoot apices of 6-month-old plantlets of WT and transgenic Jatropha. The qRT-PCR results were obtained using two independent biological replicates and three technical replicates for each RNA sample extracted from the apex of the 35S:*JcAP1* transgenic and WT shoots. Transcript levels were normalized using the *JcACTIN1* gene as a reference. The mRNA level in WT was set as the standard, with a value of 1.

**Figure S3 Flower morphological characteristics of Jatropha in different developmental stages.** (A) Inflorescence bud stage 1 (IB1): 0-5 days, inflorescence buds are visible; (B) inflorescence bud stage 2 (IB2): 1 week after IB1; (C) inflorescence bud stage 3 (IB3): 1 week after IB2; (D) flower bud stage 1 (FB1): 1 week after IB3; (E) flower bud stage 2 (FB2): male flower buds (MFB) and female flower buds (FFB) are identifiable one week after FB1; (F): male and female flower stage: male flowers (MF) and female flowers (FF) bloomed one week after FB2. In (E) and (F), red arrows indicate FFBs and FFs, respectively; and pink arrows indicate MFBs and MFs, respectively. Bars = 1 cm.
