# Supplementary figures and images for: Ectopic expression of Jatropha curcas APETALA1 (JcAP1) caused early flowering in Arabidopsis, but not in Jatropha
Source: PeerJ. 2016 Apr 25;4:e1969. doi: 10.7717/peerj.1969 (PMC4860315; doi:10.7717/peerj.1969)

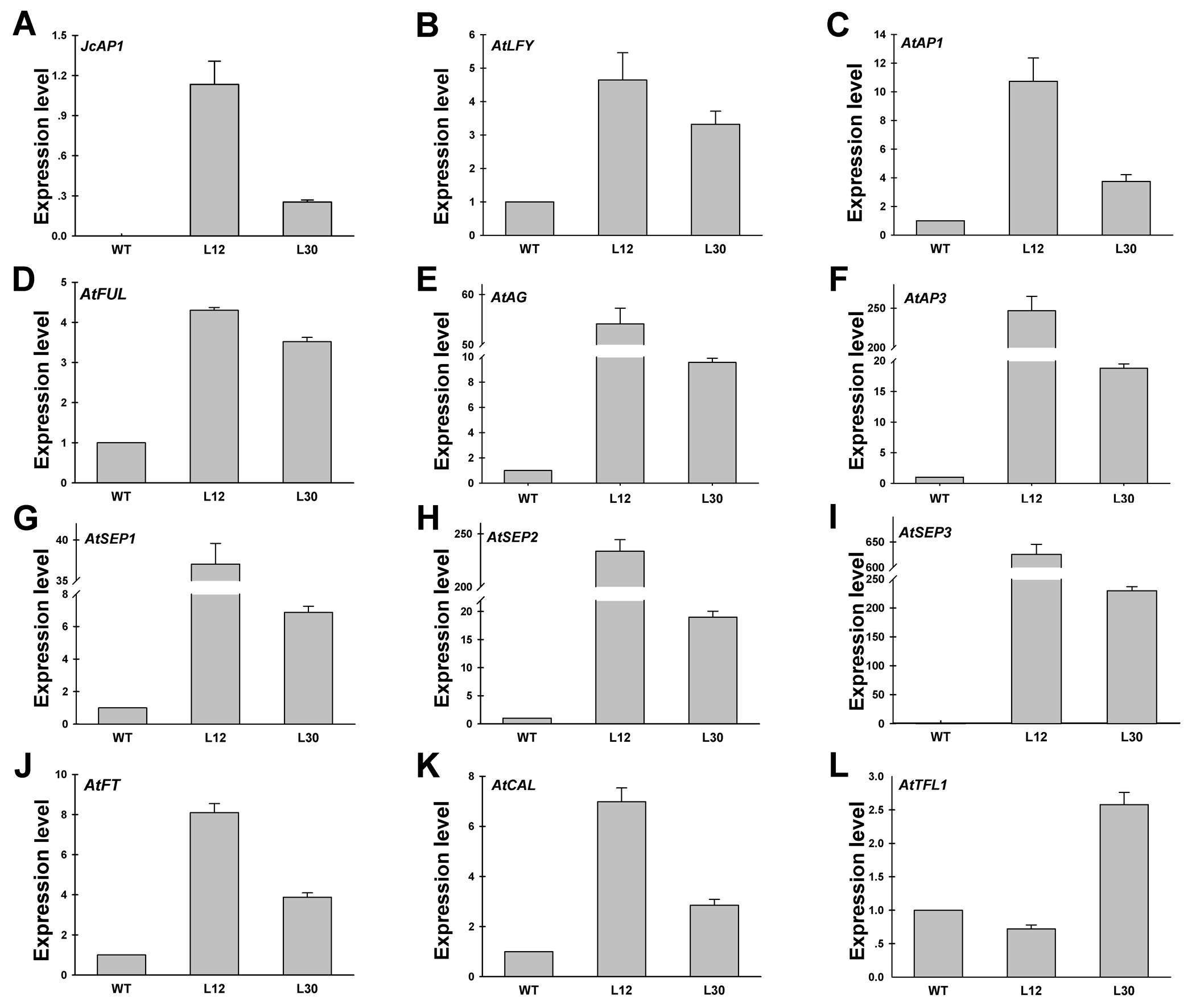

Supplement: Figure S1 — (A) The expression level of JcAP1 in WT and transgenic Arabidopsis L12 L30 plants; JcAP1 expression was not detected in WT; transcript levels were not normalized. (B–L) The expression levels of AtLFY, AtAP1, AtFUL, AtAG, AtAP3, AtSEP1, AtSEP2, AtSEP3, AtFT, AtCAL, and AtTFL1, respectively. RNA sample extracted from apex and rosette leaves of 35S: JcAP1 transgenic and WT plants cultured for 15 days in a pot. Transcript levels were normalized using the AtACTIN2 gene as a reference. The mRNA level in WT was set as the standard, with a value of 1. [file peerj-04-1969-s002.jpg]

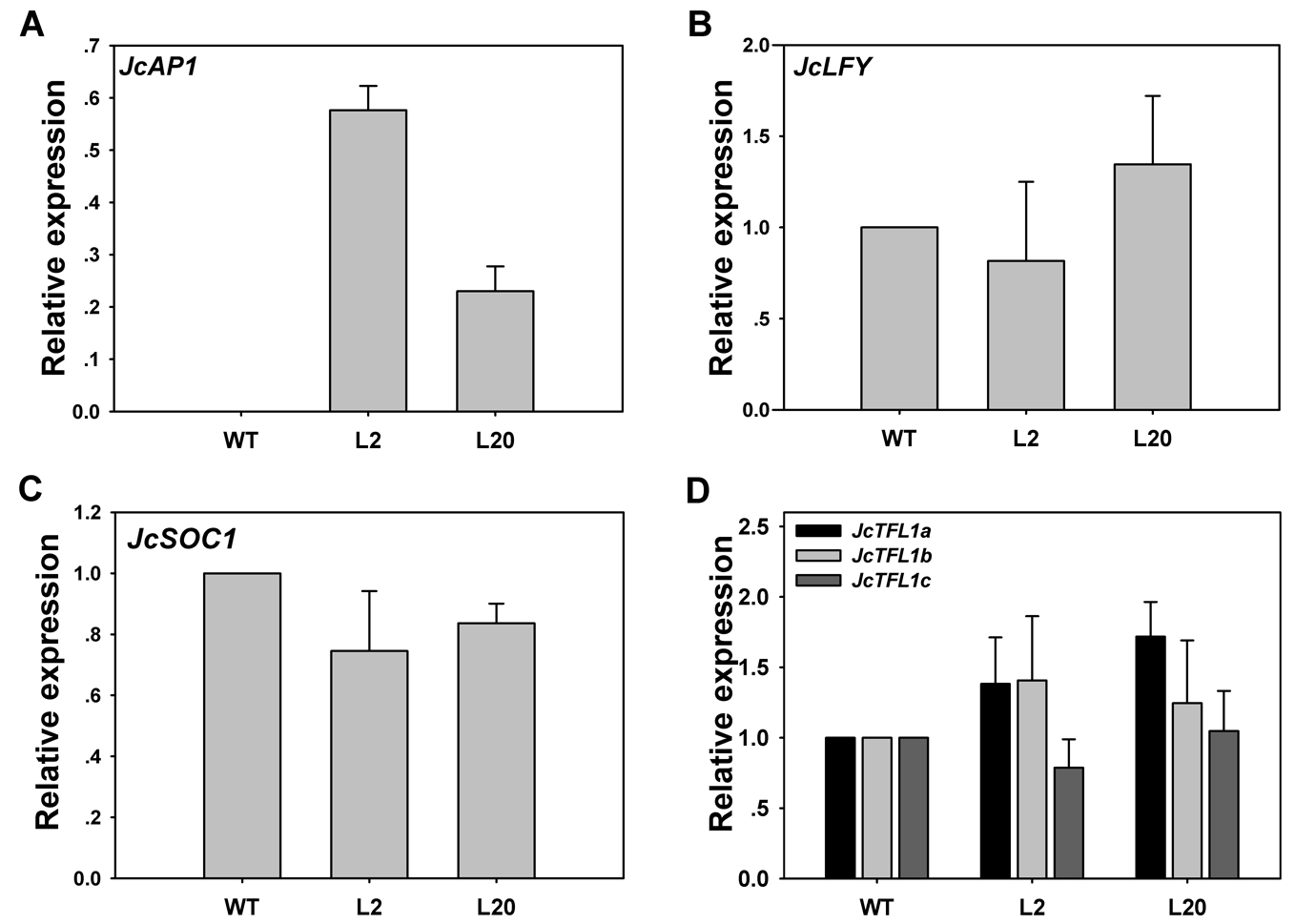

Supplement: Figure S2 — The expression levels of JcAP1, JcLFY, JcSOC1, and JcTFL1s were detected in shoot apices of 6-month-old plantlets of WT and transgenic Jatropha. The qRT-PCR results were obtained using two independent biological replicates and three technical replicates for each RNA sample extracted from the apex of the 35S: JcAP1 transgenic and WT shoots. Transcript levels were normalized using the JcACTIN1 gene as a reference. The mRNA level in WT was set as the standard, with a value of 1. [file peerj-04-1969-s003.jpg]

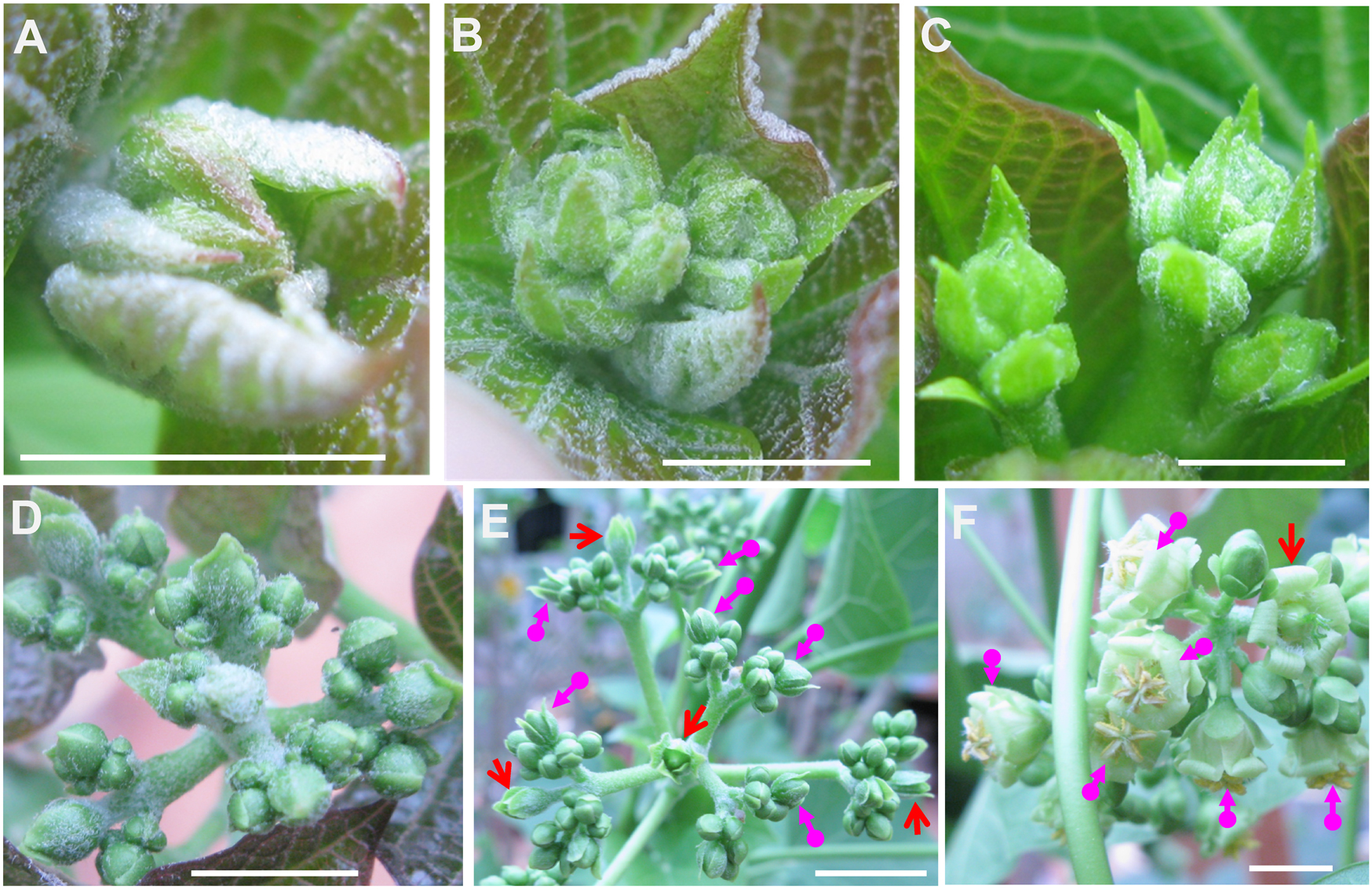

Supplement: Figure S3 — (A) Inflorescence bud stage 1 (IB1): 0–5 days, inflorescence buds are visible; (B) inflorescence bud stage 2 (IB2): 1 week after IB1; (C) inflorescence bud stage 3 (IB3): 1 week after IB2; (D) flower bud stage 1 (FB1): 1 week after IB3; (E) flower bud stage 2 (FB2): male flower buds (MFB) and female flower buds (FFB) are identifiable one week after FB1; (F): male and female flower stage: male flowers (MF) and female flowers (FF) bloomed one week after FB2. In (E) and (F), red arrows indicate FFBs and FFs, respectively; and pink arrows indicate MFBs and MFs, respectively. Bars = 1 cm. [file peerj-04-1969-s004.jpg]

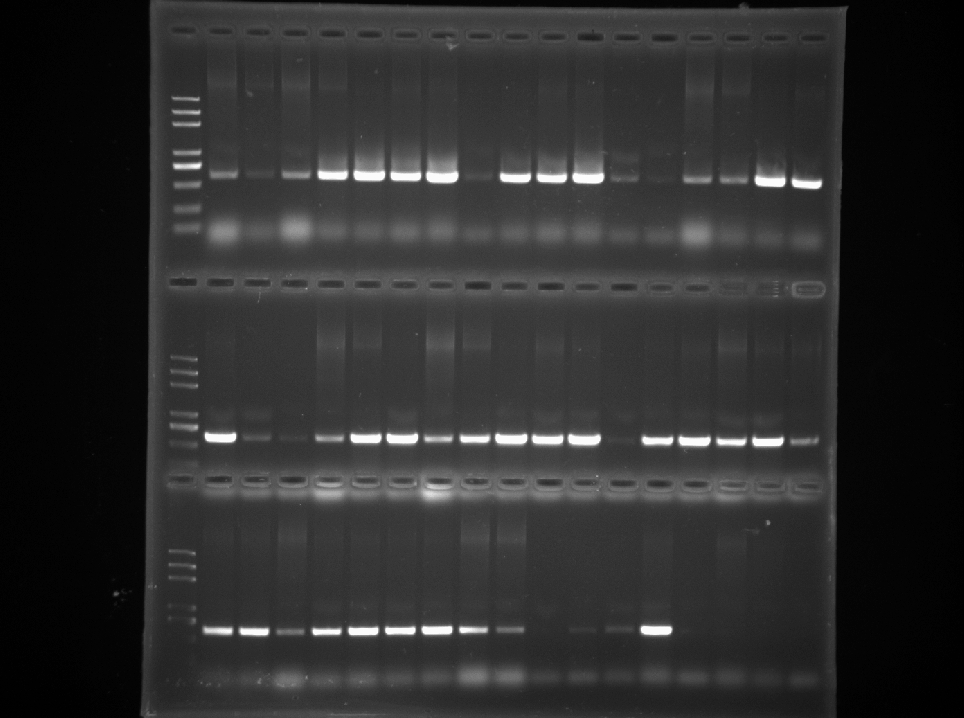

Supplement: Data S1 — Transgenic arabidopsis flowering time and gene expression levels. [file peerj-04-1969-s005.zip › raw data/pcr 2014-12-12 15hr 45min.jpg]
