# Supplementary material for: Ectopic expression of Jatropha curcas APETALA1 (JcAP1) caused early flowering in Arabidopsis, but not in Jatropha
Source: PeerJ. 2016 Apr 25;4:e1969. doi: 10.7717/peerj.1969 (PMC4860315; doi:10.7717/peerj.1969)

*AAAAATTTATACATATTTGTTGAA*

**ATG***GGTAGAGGTAGGGTTCAGTTGAAGAGAATAGAGAACAAGATCAATAGGCAAGTAACA*

**MGRGRVQLKRIENKINRQVT**

*TTTTCCAAAAGAAGGGCTGGTCTGTTGAAGAAAGCTCATGAGATCTCTGTTTTGTGTGAT*

**FSKRRAGLLKKAHEISVLCD**

*GCTGAGGTTGCTTTGATTGTTTTCTCCCACAAAGGAAAGCTCTTTGAGTACTCTACTGAT*

**AEVALIVFSHKGKLFEYSTD**

TCGTG

**S C**

gtaaatttttctttctttattctttcttcattgatattttttttatatgtatatatgcatatggtttttgtttaggttttctttctttcttttgtttaggttttatcaattattacaagatctgggtttagggaatttgtttatccattttcctcagtccacattaattttgggactttttttattattttttcaaatttaattttcttggtttcgtggctgtttcttcacagaaaaccttatccatttttcttcgtacagaacacattattgtctacatatatgcatgaaagatagatatcttgcatacagaaaaaaaaatatatttatgtatttatattatcctttttttccataaaaaatttttattttctcttgtgaaaatcagtgactgatactctaggtatcagagtttctgcttttggacttaatatatgttgcatttgaagttaaaccactatttagcatcttgaataatttaatgcagagagtttagaggctaataacatacttcataaattttttttttgaaagggactttatatgtattatcatgttctaaatatgttgttattattattattattaaagtatttcaccctcaaaacctagtcttttactaaattcagaaatgtaatttcagctttatattgattttcttttcttttcttttttaattttttatagcactatattaatctttgacataatcaaaggtgcaacacttatacttgattttgtgaaaatttataaattataataaacatatattgaaaaagatttaattgaatttactctctttttgccccatttctttttctcgcaaaagctgtggatttctacatgaaaacagatattataaaaattagtacataatattgtatggacagatttatgcaacccatcttgacatctgtctcagatccactgccaatttaactgtacttttgacttcagttgttttattgtctgcacgttatgatgttataataataataattgcatcgggagattgatttgaactctggaagaatataaatctacgatccctcaaaaggaggtccaaatttaaatgcaaaacctattaatttgattagtcttttatttggtttttaggattataccgtaactactggtttctctttcagttgtacggatttttattaaaaaatgttttattttatataattatattgagttttcatgtgtgaaattaatataattttatgataaaacacaaaaatcatttcatggttttttctgtttactttaaaataagggttattacataatggataaaatgtgcatcttagtactcacaattactttctttctaggaatgtgttctagtgcttttacttagtgaagtctttatgtaatgataacttgtgctaattattattatcatgtttttctttttccatataattaattgatgcttaacattttttgttttccgtaaatcaaaaaatatttaaatttaatataaagtttgaatctttaatctcatgcaccgcgagacagacacttgacttaatttctttgaatctcgacagatgatattcaatttgctttttattacatgtttgtctttttggataagctggtttgatgttattactgattaaatttcttggtaacttaattttaatatatgctgctatatacttccatacagtcaaaatgcttgtatggatttttattattaacaattggcttatgttattgttttaatacttagtcatgcatacttgctgcattttgctcacttttgtatctacacgacagggtagctttctccttagttcgtcaaattagtaaaaatttttataatcgagaaaactcgagtttaaaacctctcactactgtttatataagtgagaagatcaactaaatcacgtataattgacttgaatttttattcatattaattaatgttaatcatgttattagctttgcttctattgattgttttctttttttttctttttttgatactgtagattatgaggtattcttcggatagtttatgcacaccgagtgctagtgtcattagtactattattcctggagtgcgcacttcctatagtggcactgaaccctaattaataaggaaggggaattctcatctctttcatatcaactgaattgactccactggattggttcttggttgtgacttctggagaatgtgcaattttgagtatattgcttattctgttttttattttgtatatataattaacggaaaattttgtgcttattttttcaatatttcatatcaatgcctcttttgatatctaaactgcagtatgaatcatgaacaaaacaaagaaattccagtgacttgtagctgtaaaatttatgtttctcaattgccaatttgtgattccctatttgctcttattgcattatccagtgatgtttaatattctttagtccatcaaacctgttaaccaacgttgttgattactttcttgtacag

CATGGAAAAGATTCTTGAACGCTATGAGAGGTATTCTTATGCAGACAGGCAGCTC

**MEKILERYERYSYADRQL**

ATAGCAAACGATCTTAATTCACAA

**IANDLNSQ**

gtaagttctctagtacaaggacgagtttggattccttgtcccatcataaaaaattcagagtttatatatatatatatatatgaatcaaaactcagtattaatggaaccataatatataaaattagtgattgacaaatgacaaccatgaggttaattaattatatatgcag

GAGAATTGGACCCTGGAATATAACAGACTCAAGGCG

**ENWTLEYNRLKA**

AAGGTTGAGCTTTTAGAGAGAAACCATAG

**KVELLERNHR**

gtactaattattagtgaatttaattattttcttaattatctattttcctttgctgttattcaatttaatgataatattcaaacctcaacag

GCACTATTTAGGAGAAGATTTAGACTCATTG

**HYLGEDLDSL**

AGCTTGAAAGAGCTCCAAAACTTGGAGCAACAGCTTGATACTGCTCTTAAGCACATCCGA

**SLKELQNLEQQLDTALKHIR**

GCAAGAAACAACCAACTGATGCATGAAT

**ARNNQLMHE**

gtatttatataaatgcttaaatttccttttatttgatttatattaacggatagtagtaacatgtttgcttttcttgcagaaccaactgatgcatgaat

CCATCTCTGAGTATCAGAAAAAG

**SISEYQKK**

gtaatcgttaatcaaaattaacaaaaaattgctttcaagcaattgagtgatattgcaggaggaactagggtttctgctgcagtttttaattattacactgactatatacattagcattgttttcatagaatgaagaagatataatttgcttagattcggtaaaatttaattacttgatttagaacatgtctaaataagggtattagtagctcagttacagaaaatccaatggcttaattagagccagatacatacagcaatcatgttagcctgcaacataatgcacttgggcttgtttagggttttatgtgctaaatcttttgggttctaaaatttgcctaaagaaattccatagattataagcatttaaagaatgcctttagagctgaattactaatagctagttaaacttcatccttctcaaagtattgttatggacagaaaaaccctaaagtgcaggttctcttttgttttctgcttgttataaagtttgtcccagatgaagctaatcttgcgtcatgatattcttttctttttcttcttttttcatctgtgacag

GAGAAGGCA

**EKA**

ATAGAAGCGCAAAATAGTATGCTAGCAAAGCAG

**IEAQNSMLAKQ**

gtaacttatctttattattcaaaagcatacaaatcactctttccagtactacatagagaaaggttcatataagtacatagattgtgaaaattgccaattgccaagttcgattacttttttgacaatgccaaatcaaaaggcattctgcttgaagataacaggaaattacgattttcaaactggtacggcgcaattcttgcagttttatgaactttgtttcttagaacagtagaattactgagtaggattcaatatctagattacatagaagaataggttttgatagtacaggtactaaatgggtatcccactgtttcacattcctaattatagattatctgataatttttattaagaaccattctctctctatacatatatattaaataaattaatgataactacaagcttttttatttttttcttttttgtcaattgggtatctattgagttcgaactcccaatcttataatcttgagaacgacttctatgcgaaaactcataggaagagttgaatttttaggccaagtggttgaattacaacaaatttctcagtgttataagagactgcaatgagtcttcatacaaaggctattagcatacatgatagtaatatgtgtatgtgtgtatataggataattattgatgactagaaactcaagattaattagttttgtaattttgtaatag

ATTAAGGAGAAGGAGAAGGCAGTGGCA

**IKEKEKAVA**

CAGCAGGCACTTTGGGAGCAGCAAAACCGTAACACCCATATGTCACCCTTCCTTGTGCCA

**QQALWEQQNRNTHMSPFLVP**

CAGCAACCACTTCCTTGTCTAAATATTGG

**QQPLPCLNIG**

gtaatccatttttaatttgcttgcttttatattaatgacttcttagaatatacacatatttccactgtctaattatttgacatgtgatattacttatgtgcag

TGGCACTTACCAGGAAGAAGCAGTACCAGAA

**GTYQEEAVPE**

GTGAGGAGAAATCAGCTGGACCTAACATTGGAACCAATATATTCATGTCATCTTGGATGC

**VRRNQLDLTLEPIYSCHLGC**

TTCACCACA***TAA****TTATCAATTTGGTCGAAGAAATTAATTAAAATGGAAAACAGGAGAGAG*

**FTT-**

*ATTACTACAT*

**Figure5. *JcAP1-B* genomic DNA sequence, cDNA sequence and amino acids sequence .** The introns were shown in lower case letters; cDNA sequence were shown in capital letters; amino acids were shown in bold capital letters; untranslated region were shown in italic capital letter

>JcAP1

MGRGRVQLKRIENKINRQVTFSKRRAGLLKKAHEISVLCDAEVALIVFSHKGKLFEYSTDSCMEKILERYERYSYADRQLIANDLNSQENWTLEYNRLKAKVELLERNHRHYLGEDLDSLSLKELQNLEQQLDTALKHIRARNNQLMHESISEYQKKEKAIEAQNSMLAKQIKEKEKAVAQQALWEQQNRNTHMSPFLVPQQPLPCLNIGGTYQEEAVPEVRRNQLDLTLEPIYSCHLGCFTT

The vector construct in this study:


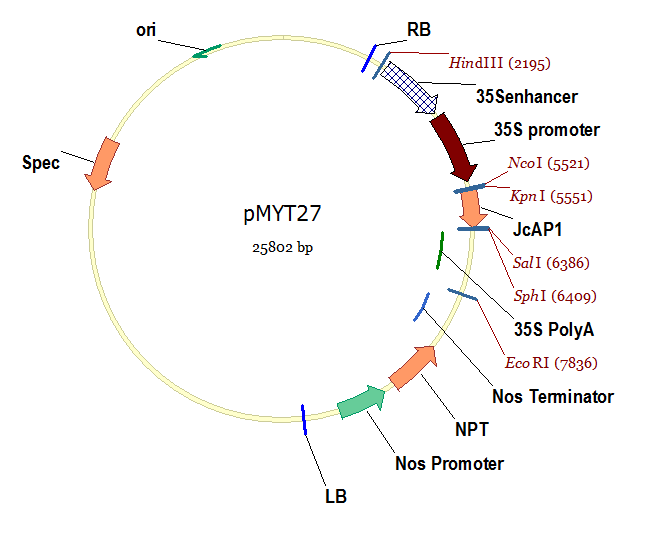

Supplement: Data S1 — Transgenic arabidopsis flowering time and gene expression levels. [file peerj-04-1969-s005.zip › raw data/JcAP1 gDNA squence.docx]
